# Characteristics and associated factors of health information-seeking behaviour among patients with inflammatory bowel disease in the digital era: a scoping review

**DOI:** 10.1186/s12889-024-17758-w

**Published:** 2024-01-27

**Authors:** Zijun Ni, Lingli Zhu, Shuyan Li, Yuping Zhang, Ruiyi Zhao

**Affiliations:** 1 Nursing Department, The Second Affiliated Hospital of Zhejiang University School of Medicine, No.88 Jiefang Road, Hangzhou, 310009 China; 2 Department of Nursing, School of Medicine, Zhejiang University, Hangzhou, China

**Keywords:** Inflammatory bowel disease, Health information seeking behaviour, Review

## Abstract

**Background:**

Health Information-Seeking Behaviour (HISB) is necessary for self-management and medical decision-making among patients with inflammatory bowel disease (IBD). With the advancement of information technology, health information needs and seeking are reshaped among patients with IBD. This scoping review aims to gain a comprehensive understanding of HISB of people with IBD in the digital age.

**Methods:**

This scoping review adhered to Arksey and O'Malley's framework and Preferred Reporting Items for Systematic Reviews and Meta-Analyses extension for Scoping Reviews frameworks (PRISMA-ScR). A comprehensive literature search was conducted in PubMed, Embase, Web of Science, PsycINFO, CINAHL, and three Chinese databases from January 1, 2010 to April 10, 2023. Employing both deductive and inductive content analysis, we scrutinized studies using Wilson's model.

**Results:**

In total, 56 articles were selected. Within the information dimension of HISB among patients with IBD, treatment-related information, particularly medication-related information, was identified as the most critical information need. Other information requirements included basic IBD-related information, daily life and self-management, sexual and reproductive health, and other needs. In the sources dimension, of the eight common sources of information, the internet was the most frequently mentioned source of information, while face-to-face communication with healthcare professionals was the preferred source. Associated factors were categorized into six categories: demographic characteristics, psychological aspects, role-related or interpersonal traits, environmental aspects, source-related characteristics, and disease-related factors. Moreover, the results showed five types of HISB among people with IBD, including active searching, ongoing searching, passive attention, passive searching, and avoid seeking. Notably, active searching, especially social information seeking, appeared to be the predominant common type of HISB among people with IBD in the digital era.

**Conclusion:**

Information needs and sources for patients with IBD exhibit variability, and their health information-seeking behaviour is influenced by a combination of diverse factors, including resource-related and individual factors. Future research should focus on the longitudinal changes in HISB among patients with IBD. Moreover, efforts should be made to develop information resources that are both convenient and provide credible information services, although the development of such resources requires further investigation and evaluation.

**Supplementary Information:**

The online version contains supplementary material available at 10.1186/s12889-024-17758-w.

## Background

Crohn’s disease (CD) and ulcerative colitis (UC) are the two most common forms of inflammatory bowel disease (IBD), which is a complex chronic disease increasing worldwide [1]. IBD is believed to result from a combination of genetic, gut microbiome, and environmental factors, including diet [2]. Patients with IBD experience a wide range of symptoms, such as abdominal pain, chronic and recurrent diarrhoea, fatigue, and more, which severely affect their health and quality of life [1]. Due to the large heterogeneity of IBD and the diversity of treatments, modern management of IBD should be a joint decision between informed patients [2, 3]. Health information seeking has become a common phenomenon for people with IBD during participation in medical decision-making and self-management of the disease. In the field of information science and chronic disease research, previous studies have demonstrated that a person’s health information-seeking behaviour has the potential to positively influence the process and outcomes related to coping with or adjusting to an illness or condition, such as improving treatment adherence and self-management abilities [4, 5]. However, it can also have adverse effects, such as contributing to health anxiety (e.g., Cyberchondria), intensifying doctor-patient conflicts, and leading to excessive healthcare utilization [6–8]. Furthermore, patients seeking health information may encounter conflicting information from various sources, such as the internet, expert opinions, reputable medical websites, and anecdotes [9]. Reviewing and reconciling these conflicting pieces of information can exacerbate patients’ medical decision-making conflicts, ultimately influencing their treatment choices and outcomes [9, 10]. Therefore, it is vital to thoroughly understand the health information-seeking behaviours of patients with IBD, which could help health and information services enhance and facilitate their access to trustworthy information.

Health information-seeking behaviour (HISB) has been described as the purposeful seeking of information related to an individual's health, including health promotion activities, risk factors, and illnesses [11, 12]. In an extensive conceptual analysis of HISB by Zimmerman and Shaw [13] in 2020, the characteristics of HISB were divided into an information dimension and a method dimension, i.e., the types and amounts of health-related information sought, the specific actions implemented to obtain the information, and the sources used by individuals. The rapid development of information and communication technologies (ICTs) has recently heightened health information demand and expectations among consumers, constantly reshaping their HISB [14]. Given the diversity, accessibility, immediacy, and interactive nature of ICTs, they have expanded the breadth and depth of information available to patients and improved access to health information sources, such as mobile devices, websites, and social media [15]. It has captured the attention of many researchers, and many empirical studies have investigated the information needs and sources of information for people with IBD during this technologically advanced period [16–18]. However, to our best knowledge, few scoping or systematic reviews have directly addressed the HISB of patients with IBD and synthesised this body of knowledge in the digital era. Al Khoury et al. [19] systematically reviewed perspectives and expectations of patients with IBD. Although the systematic review demonstrated that patients with IBD expected more information about their disease process, shared decision-making, and symptom control, it provided limited details on their HISB, including the discretionary actions employed to get information and relevant variables. A scoping review of the evolution of perceived engagement and care needs of patients with IBD across the life-cycle also only briefly mentioned their information needs, lacking an in-depth analysis of characteristics and associated factors of HISB in the digital era [20]. Hence, it is necessary to conduct a scoping review of the HISB among patients with IBD during this technologically advanced period to inform better health information system designs and ensure better patient information services. In this scoping review, we aimed to examine the state of research on the HISB among patients with IBD and reveal the information and methodological characteristics and associated factors of HISB in the age of information.

## Methods

This scoping review was undertaken in line with Arksey and O’Malley’s framework for scoping studies [21] and the Preferred Reporting Items for Systematic Reviews and Meta-Analysis Extension for Scoping Reviews (PRISMA-ScR) guidelines [22]. The purpose of this review is to provide our readers with an overview of how HISB among IBD patients has been studied and present implications for future research. While we have formulated four research questions to guide the review and provide structure, it is important to note that these questions serve as a framework to explore the various aspects of HISB among IBD patients, rather than to answer a specific and focused research question or to critically evaluate the available evidence. Hence, a scoping review was conducted instead of a systematic review [21, 23].

### Identifying the research question

Based on the information and methodological characteristics of HISB, this review was guided by the following questions:

1. What are the health-related information needs of patients with IBD?
2. Where do they seek health information?
3. What are the types of health information-seeking behaviour of people with IBD?
4. What factors are associated with the HISB of patients with IBD?

### Search strategy

The comprehensive literature search was conducted in PubMed, Embase, Web of Science, PsycINFO, and CINAHL. Additionally, Chinese sources were also searched: China Biology Medicine Disc (CBMdisc), China National Knowledge Infrastructure (CNKI), and China Wanfang Database. Based on a preliminary review of the literature and our clinical and research experience, our search strategy combined the mesh subject headings (MESH) and a series of free-text terms for the following key terms: information seeking behaviour, help seeking behaviour, information sources, information needs, patient Education as topic, information preference, Crohn’s disease, ulcerative colitis, and inflammatory bowel disease. Specific search terms were modified for each database (See Supplementary material 1). Besides, studies published from January 1, 2010 to April 10, 2023 were considered. Because this period witnessed revolutionary advancements in ICTs including mobile devices and social media developments [24, 25], and there is evidence that the majority of studies on the use of social media in healthcare were published after 2010 [26, 27]. To uncover supplementary research, we manually scrutinised the reference lists of the incorporated studies and relevant papers [21, 23]. The research team devised the search strategies, and the first author conducted the searches accordingly.

### Eligibility criteria and study selection

The inclusion criteria for the studies were (1) peer-reviewed journal articles; (2) observational studies considering HISB of patients with IBD; (3) studies published in English or Chinese language. Studies were excluded for any one of the following: (1) reviews, comments or opinions, editorials, study protocols with no empirical data, or intervention studies; (2) included patients ≤ 18 years old, which groups are likely to have special HISB as with other paediatric patients with chronic diseases; (3) focused on the HISB of healthcare professionals (HCPs); (4) focused on analysing the quality of information on the Internet or social media; (5) studies with critical data missed, or full text unavailable.

Identified records were imported into Endnote X9 to form a single combined library. Of the 3,612 articles after deletion of duplicates, three reviewers (ZN, SL, and LZ) screened the first 200 articles to ensure a consistent understanding of the eligibility criteria [28]. Subsequently, two primary reviewers (ZN and LZ) independently screened the collated titles and abstracts. To avoid missing potentially pertinent studies, we did not pre-emptively eliminate papers that appear to focus on the characteristics or value of information sources at the title and abstract screening stage. The full texts of the remaining studies were retrieved and dependently screened for their eligibility by two reviewers (ZN and LZ), with any disputes resolved by a third reviewer (SL). Following the scoping review methodology, we did not perform any formal quality assessment on the studies in this review [21, 22].

### Data extraction and synthesis

Two independent reviewers (ZN and LZ) manually extracted data from the included studies into Excel and summarized it in a tabular format. According to the objectives and review questions of this study, data for each study was extracted as follows: the first author, title, country, year of publication, study design, methodology, sample size, aims, health information needs, types of HISB, information sources, and associated factors. In case of disagreement between the pair, ZN and LZ reviewed the articles again and consulted with SL. Study authors were contacted for details when the necessary information was missing or incomplete.

We performed a content analysis of the included studies both deductively and inductively, because it leads to an enrichment of the understanding of the studied object [29–31]. In other words, we were receptive to new themes. In phase 1, in addition to transferring the data extracted to a data extraction form, the extracted data were uploaded into NVivo12. In NVivo12, the uploaded data were deductively coded into pre-defined main themes that matched the review questions and, thus, focused on results relating to health information needs, information sources, the types and associated factors of HISB. In phase 2, two reviewers (ZN and LZ) re-examined and synthesized in an inductive approach captured in the data within each theme, and identified sub-themes. In addition, in the themes of intervening variables and types of HISB, we further mapped the related sub-themes into Wilson's model of information behaviour [32]. To ensure rigor and minimize biases, the study team extensively repeated and re-evaluated the identified categories several times [33].

Wilson’s model of information behaviour depicts the information cycle, from information need to information use, and includes intervening variables and mechanisms significantly influencing information behaviour [32, 34]. Four different types of information-seeking behaviour are identified: passive attention, passive search, active search, and ongoing searching [32]. The first two are passive modes, and the last two are opposite. Passive attention refers to obtaining information without deliberately seeking it, such as through watching television or listening to the radio [32]. Passive search is a search for other types of information that happens to be relevant to the individual [32]. This exposure to relevant information through passive search often triggers an active search, where the individual actively seeks out additional information on the topic of interest. And it is the "primary mode" of information seeking [32]. Ongoing searching, another of the active modes, occurs when individuals periodically update or expand their knowledge framework after an active search has established the basic framework [32]. Additionally, Wilson categorised the intervening variables into five groups, including psychological, demographic, role-related or interpersonal, environmental, and source characteristics [32, 34].

## Result

### Study characteristics

**Fig.1:**
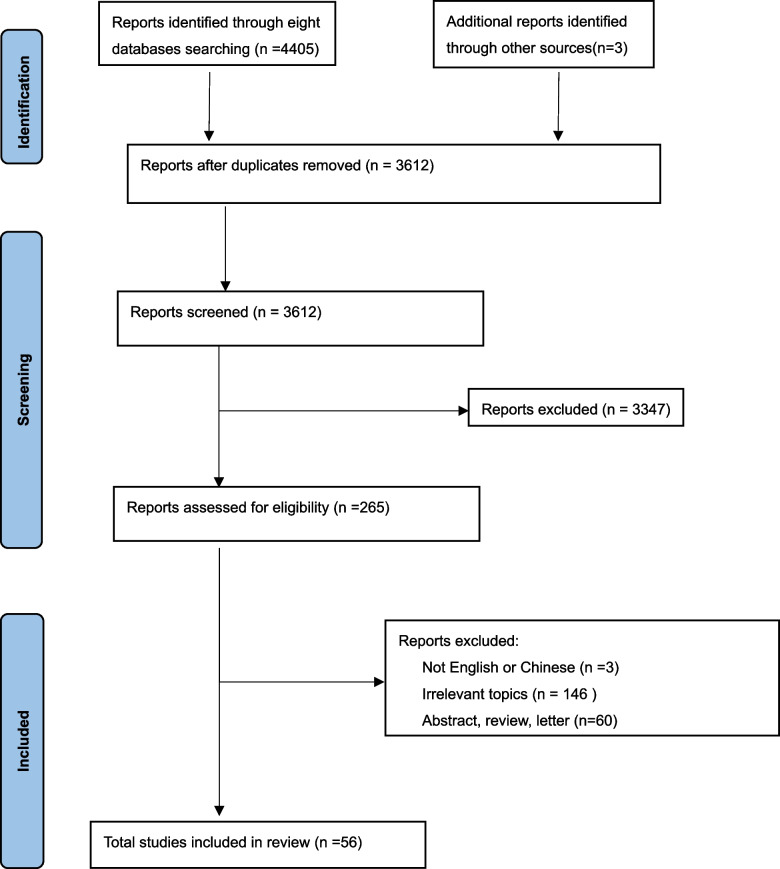
PRISMA flow diagram

In our preliminary search, a total of 4408 records were identified. After removing duplicates and screening, the final sample consisting of 56 studies was obtained (See Fig. 1), including six studies in Chinese [35–40]. Of all the studies, over half (*n* = 32, 57.14%) were published within the last five years, especially in 2021 (*n* = 10,17.9%). The included studies originated from 19 countries (based on the first author’s affiliations), with the majority from Europe (*n* = 28, 50%). A significant portion of the included studies (*n* = 34, 60.71%) employed quantitative approaches, while 17 (30.36%) utilised qualitative approaches, and 5 (8.93%) were based on mixed methods designs. As for specific methods, surveys were the primary choice (*n* = 32, 57.14%), followed by interviews or focus groups (*n* = 17, 30.36%). Interestingly, there are three studies analysing forum posts [41–43]. Regarding data types, all studies were based on cross-sectional data, and 5 studies (8.92%) utilized multicenter data. Participant sample sizes in the included articles varied widely, ranging from 13 [35] to 3,115,477 [44]. Four studies did not specify the sample size due to their methodology [41–43, 45]. Among the 44 studies (78.57%) involving participants with both Crohn's disease (CD) and ulcerative colitis (UC),12 (21.43%) also included participants with unspecified or other types of IBD diagnoses. Additionally, six studies (8.93%) focused on CD (*n* = 4) [18, 46–48] or UC (*n* = 2) [49, 50]. Among the final included studies, only a few (*n* = 2) examined HISB as a whole variable [16, 51], while the majority (*n* = 41) focused on one or two aspects of HISB, such as content of information needs, information sources, and types of seeking behaviour [13]. Approximately 73.21% (*n* = 41) of the studies discussed various information needs of patients, and 91.07% (*n* = 51) investigated patients' information sources. However, types of HISB based on Wilson's model were identified in only 67.86% of the studies (*n* = 38). (The overview of the included studies is presented in Supplementary material 2).

### Content of health information needs

We identified five main categories related to information needs from 46 studies [16–18, 35–43, 45–78], reporting on a total of 19 information needs (see Table 1). IBD treatment information (*n* = 39) was the most frequently mentioned category, primary focusing on medication details for patients with IBD. From the 16 studies that reported medicine-specific information needs, we noticed that patients with IBD were primarily focused on information about medication options [16, 42, 48, 51, 52, 55, 60, 63, 73, 74, 76, 77] and potential side effects of drugs [16, 18, 48, 51, 52, 54, 55, 62, 65, 73, 74, 76, 77]. Moreover, seven studies focused on specific information about surgery, including surgical procedures [46, 47, 50, 56, 57, 70], surgical complications, long-term recovery and practical matters such as returning to exercise, dietary restrictions, management of stomas, and cosmetic issues after surgery [46, 47, 49, 50, 57, 70]. Regarding information about alternative treatment, some studies indicated that certain patients expressed an interest in knowing the availability of non-conventional therapies for IBD and how these therapies can support their treatment [16, 74]. Other important categories of information needs identified included daily life and self-management (*n* = 36, 64.29%), IBD-related basic information (*n* = 33, 58.93%), and sex and reproductive health (*n* = 15,26.79%). Furthermore, We grouped some other information content into the miscellaneous category, including health insurance policy, medical resources, and research on new medicine [16–18, 35, 37, 39, 41, 45, 48, 51, 54, 55, 58–60, 62, 64–66, 68, 71, 73–75, 77]. And during the COVID-19 pandemic, two studies showed that they were more interested in information about how to deal with COVID-19 [58, 79].

**Table 1:** Information needs categories of patients with IBD

Main category	Subcategory	Number of studies	Studies
**IBD-related basic information**	Generalities about the disease	10	[16, 36, 37, 40, 43, 51, 60, 69, 72, 74]
	Common symptoms /Extraintestinal manifestation	21	[16, 35, 37, 39, 41–43, 45, 48, 51, 52, 55, 59, 60, 66, 68, 73–75, 77, 78]
	Disease complications	13	[35, 39, 41, 48, 51, 52, 55, 63, 66, 69, 75–77]
	Etiology and mechanism	12	[35, 36, 41, 48, 51, 52, 55, 60, 63, 68, 69, 77]
	Prognosis/ Risk of cancer	9	[16, 35, 48, 51, 52, 54, 73, 76, 77]
	Tests and diagnosis	7	[16, 37, 55, 69, 72, 73, 78]
	Vaccination	3	[16, 51, 78]
**Treatment**	Medication	33	[16–18, 35–43, 45, 48, 51, 52, 54, 55, 60, 62, 63, 65, 66, 68, 69, 71–78]
	Surgery	21	[16, 36, 37, 41, 46–52, 56, 57, 68–70, 72, 73, 76–78]
	Alternative treatments	8	[16, 17, 36, 43, 48, 51, 55, 74]
**Daily life and self-management**	Diet and nutrition	29	[16, 18, 35–37, 39–41, 43, 45, 48, 51, 52, 54, 55, 60, 62, 63, 65, 66, 68, 69, 71–74, 76–78]
	Coping with IBD (e.g., common self-care skills)	22	[16–18, 35, 37, 38, 42, 48, 51, 52, 54, 55, 59, 62, 63, 68, 69, 71, 72, 74, 75, 77]
	Exercise	6	[35, 39, 55, 62, 73, 74]
	Issues in work or learning	10	[16, 39, 48, 52, 54, 66, 73, 74, 76, 77]
	Psychological aspects	11	[16, 37–39, 45, 51, 55, 65, 66, 74, 75]
	Experience of other patients	5	[16, 18, 51, 59, 63]
	Travel-related health issues	3	[53, 67, 73]
**Sex and** **reproductive health**		15	[16, 36, 37, 39, 47, 48, 51, 52, 55, 61, 66, 72–74, 76]
**Miscellaneous needs **^**a**^		27	[16–18, 35, 37, 39, 41, 45, 48, 51, 52, 54, 55, 58–60, 62, 64–66, 68, 69, 71, 73–75, 77]

^a^This includes, for example, health insurance policy, medical resources, and research on new medicine

In this scoping review, we found that many of the 46 studies (*n* = 21) prioritized patients' information needs without setting specific scenarios (such as focusing on surgery or pregnancy), and Table 2 presented the extracted top four ranked needs from each study. Medication information (*n* = 17) was identified by people with IBD as the most critical and necessary need out of the 19 information needs stated above, followed by diet and nutrition (*n* = 13) and generalities about the disease (*n* = 9).

**Table 2:** Prioritization of information needs

Information needs	Number of studies	Studies
Medication information	17	[16, 17, 36, 37, 39, 51, 52, 55, 65, 66, 68, 72–74, 76–78]
Diet and nutrition	13	[18, 36, 37, 39, 52, 55, 66, 68, 69, 72, 76–78]
Generalities about the disease	9	[16–18, 37, 51, 52, 69, 72, 74]
Coping with IBD	6	[17, 39, 51, 52, 55, 77]
Disease complications	6	[39, 48, 59, 66, 69, 76]
Prognosis/ Risk of cancer	6	[18, 36, 48, 72, 73, 77]
Common symptoms / Extraintestinal manifestation	6	[48, 68, 69, 73, 74, 78]
Etiological and mechanism	3	[36, 48, 76]
Surgery	2	[65, 73]
Test and diagnosis	2	[37, 55]
Alternative treatments	2	[55, 74]

### Health information sources

The source of information is one of the frequently reported aspects of information in HISB studies [80]. Of all the included studies, 91.07% (*n* = 51) investigated the current sources of information for patients with IBD, and showed the most common sources were HCPs (*n* = 45) and online (*n* = 49). Further, we also made an in-depth analysis of the online information sources of patients due to their variety in the age of information. The majority of studies (*n* = 27) used the general internet as a whole to represent all online health information source. Social media (e.g., Facebook, Twitter) and online patient communities (*n* = 19) emerged as the most widely used online sources [60, 81]. Health websites (*n* = 15), owned by educational, commercial, governmental, and nonprofit organizations, were the third most commonly used source. Moreover, general search engines (e.g., Google, Wikipedia, NHS choices) were also frequently mentioned (*n* = 9). Only five articles mentioned the use of mobile internet services by people with IBD [17, 46, 60, 65, 68]. Notably, 32 studies also investigated where patients would most prefer to seek information. Face-to-face discussion with HCPs was the most frequently mentioned preferred source of information, followed by search engines (See Table 3).

**Table 3:** The current and preferred sources of information

Information Sources		Number of studies	Studies
**Current** (*n* = 51)	Healthcare professionals ^a^	45	[16–18, 35, 36, 38–41, 44, 46–58, 60, 64, 65, 67–69, 71, 73–79, 81–88]
	Specialist printed informational materials ^b^	31	[16–18, 35, 36, 38, 39, 46–48, 50–54, 56, 58, 62, 67, 68, 71, 73, 75, 77–79, 82, 83, 85, 86, 88]
	Government organizations /Associations / Support groups ^c^	23	[16, 17, 41, 47, 48, 50–53, 58, 60, 68, 69, 73, 74, 77–79, 81, 83, 84, 86, 88]
	Other patients	18	[18, 35, 36, 38, 40, 41, 50, 56–58, 65, 68, 69, 71, 74, 79, 83, 87]
	Family members/Friends	14	[16, 17, 46, 48, 52, 53, 56, 58, 65, 67, 77, 79, 85, 86]
	Broadcast media (e.g., TV, radio)	7	[16, 38, 51, 56, 58, 79, 86]
	Lectures or meetings	3	[39, 40, 88]
	Others ^d^	3	[53, 65, 67]
	General Internet	27	[16, 35, 36, 38, 40, 44, 47, 48, 51, 53, 56, 57, 62, 64, 65, 67, 68, 71, 73, 75, 76, 78, 82, 83, 85–87]
	Social media or online communities ^e^	20	[17, 18, 38, 43, 46, 47, 50, 52, 56, 58–60, 63, 69, 73, 77, 79, 81, 82, 84]
	Health Website ^f^	16	[37, 43, 45, 46, 50, 52, 54–56, 59, 69, 77, 79, 81, 84, 88]
	Search engines ^g^	9	[17, 18, 46, 50, 55, 59, 69, 74, 88]
	Mobile internet services	5	[17, 46, 60, 65, 68]
**Preferred** (*n* = 32)	Face-to-face discussion with HCPs	24	[16, 18, 36, 38, 39, 46–48, 50–54, 65, 68, 69, 73, 77, 79, 82, 85–88]
	Search engines	4	[17, 59, 67, 78]
	An HCP-guided social media network	2	[55, 60]
	Health Website	2	[74, 84]

^a^For example, IBD specialists or nurses, gastroenterologists, surgeon, pharmacists, and family doctors

^b^For example, specialist books, brochure, magazines, patient leaflets

^c^For example, world health organization, health departments, the China Crohn’s & Colitis Foundation *CCCF*, the Crohn's & Colitis Foundation *CCFA*, and the centers for disease control and prevention *CDC*

^d^Including, for example, religious leader and travel clinic

^e^For instances, Facebook, Twitter, Instagram and YouTube

^f^Including, for example, the CCFA website, Crohn’s and Colitis Canada website

^g^For instances, Google, Wikipedia, and NHS choices

### Types of HISB of patients with IBD

In these 38 studies showing the actions taken by patients with IBD to seek information, some indications were derived from the sources consulted and the patients quoted in the qualitative studies. We analysed the information to identify five types of HISB in people with IBD, four of which deductively mapped to the Wilson’s model. The results presented that active searching (*n* = 35) was the most mentioned types in included studies. Patients with IBD actively sought information from their HCPs and various online sources, including reputable health websites, general search engines, and social media [40, 56, 62]. Notably, we found that social information seeking was a typical feature of active searching among patients with IBD over the past decade. They regularly browsed, searched, and shared information about the disease through social networks and interactions with others (e.g., friends, colleagues, experts, and online communities) [42, 50, 56, 58, 62]. Many studies (*n* = 15) revealed that patients would occasionally or continuously search to gain knowledge about self-care or the latest research developments, which is called ongoing searching [11, 62]. Five studies displayed passive attention behaviour among some patients, where they inadvertently received information from their environment, such as through the radio or television [38, 40, 56, 79, 86]. Correspondingly, seven studies noted passive searching behaviour, whereby patients accessed relevant health information through regular activities like reading newspapers or magazines [17, 35, 63, 71, 83, 86, 88]. In addition to the four types of information-seeking behaviour in Wilson's model, it was observed that certain patients exhibited a lack of engagement in information-seeking behaviour or avoided seeking information [38, 51, 70, 75, 82] (See Table 4).

**Table 4:** The types of health information-seeking behaviour

Types	Example	Studies
Passive attention	"When I first found out about Crohn's disease, I seen the commercial on TV, and it was talking about the symptoms as far as having it. I'm like, dang, I go through the same thing. " [56]	[38, 40, 56, 79, 86]
Passive search	"Information can be obtained through regular behaviours such as reading newspapers or magazines." [17]	[17, 35, 63, 71, 83, 86, 88]
Active search	"On the stoma sites, a lot of people do Vlogs, so I’ve watched them before. There’s some good ones that are helpful. " [47]	[16–18, 38, 40, 42–44, 47, 50–58, 60, 62–64, 67, 68, 70, 71, 75, 78, 81–86, 88]
Ongoing search	"I am on Facebook daily, so every now and then I see an article about IBD that catches my eyes, I’ll click on it and read it" [62]	[17, 38, 40, 42, 44, 50, 54, 56, 59, 62, 68, 71, 78, 81, 85]
Avoid seeking	"I had joined some patient groups before, and I was so shocked by the information in them that now I just don't want to know, I reject all of them." [38]	[38, 51, 70, 75, 82]

### Associated factors of HISB

In our review, we grouped and summarised the main factors influencing any aspect of HISB from the 43 included studies into six categories (See Table 5). Five categories were in line with Wilson’s model, and an additional category was disease-related factors.

**Table 5:** Associated factors of HISB among patients with IBD

Associated variables	Number of studies	Studies
**Demographic**		
Age	17	[16, 17, 36, 43, 44, 48, 50, 59, 60, 65, 66, 69, 70, 73, 76, 81, 84]
Education level	8	[17, 38, 52, 55, 60, 69, 73, 84]
Gender	11	[16, 17, 44, 48, 52, 60, 65, 68, 74, 77, 81]
Race / Ethnicity	3	[16, 61, 84]
Income	1	[55]
**Psychological**		
Disease-related anxiety and fears	6	[16, 38, 56, 69, 70, 88]
Individual interest/preference	7	[44, 53, 55, 58, 68, 70, 84]
Self-efficacy in learning	1	[49]
Self-efficacy in health	2	[44, 70]
Experience of seeking health information	4	[44, 63, 70, 84]
**Role-related or interpersonal**	4	[48, 61, 68, 70]
**Environmental**		
Place of residence	4	[36, 48, 85, 86]
**Source-related characteristics**		
**Credibility**		
Trustworthiness	7	[16, 17, 55, 60, 81, 84, 88]
Output quality	7	[17, 38, 58, 60–62, 77]
** Usefulness**		
Perceived usefulness of health information	3	[58, 60, 61]
Perceived usefulness of ways to access information	7	[45, 47, 56, 62, 71, 77, 84]
Perceived negative of health information	2	[56, 82]
**Disease-related**		
Disease duration	8	[38, 48, 66, 69, 71, 75, 78, 84]
Disease activity / Severity	8	[16, 37, 38, 51, 70, 75, 81, 84]
Experience of treatment	3	[16, 73, 81]
Types of IBD	4	[16, 72, 76, 81]

Demographic characteristics were the most often reported associated factors. Studies showed that older patients used the Internet less often to find health information than younger patients [36, 59, 60, 64]. Some studies showed that women and patients with higher levels of education might have higher information needs and search for information more frequently [17, 52]. Furthermore, Reich et al. identified that black patients are more likely to use social media to access information and manage disease than white patients [84]. One study showed that individual income was significantly associated with information-seeking content [55].

Within the psychological aspects, the frequently mentioned factors were disease-related anxiety and fears [38, 69], individual preference [55, 58], and the experience of seeking health information [44, 63, 70, 84]. It was clear that disease-related anxiety and fear would both promote information seeking and lead to information-avoidance behaviour [16, 38, 56]. Some studies identified that frequent internet users were more likely to report using ICTs to seek information of interest [44, 84]. In addition, feelings during the information seeking process, such as being empathetic to the information and too time-consuming, could affect the patient's motivation to seek and source selection [63, 84].

Associated factors of the category ‘role-related or interpersonal’ were identified in four studies [48, 61, 68, 70], focusing on characteristics linked to an individual's social roles, interpersonal relationships, and group dynamics. Childbearing women, young patients, and patients with plans to have children have significantly higher information demands concerning fertility [48, 61]. A family history of IBD is associated with a higher preference for information [68]. Four studies addressed the category of ‘environmental aspects’ [36, 48, 85, 86]. In a survey from China, it was pointed out that patients may also pay attention to the usefulness and effectiveness of Chinese medicine or acupuncture [36]. And a multicentre, cross-sectional cohort study stated that significantly more patients in Eastern Europe independently searched for additional information regarding their disease than in Western Europe [85].

Another frequently stated category was source-related characteristics, including credibility and usefulness. The higher trustworthiness of sources and the better output quality might change patients’ HISB [17, 57, 60]. Moreover, patients with IBD would like to continue their search if they perceived the usefulness and importance of health information [58, 60]. Conversely, they might avoid or refuse information seeking when they perceived negative information [82]. Many studies indicated the usefulness of ways to access information could change patients’ choices of information sources [45, 47, 62, 81].

Disease-related factors were the common variables linked to their HISB. Disease duration and severity or activity of IBD were the two most commonly mentioned variables. Studies indicated that recently diagnosed patients and patients in the active stage had higher information needs and tended to search more actively for information [52, 54]. In contrast, patients in remission were mainly interested in recent advances in research and long-term disease evolution [16]. Moreover, patients using different medications sought out different information content [54, 66, 70, 73]. There appears to be a correlation between types of IBD and health information seeking [16, 72, 76, 81]. One study stated that patients with CD were significantly more concerned about disease-related information [72], while Pittet et al. found that UC patients expressed more demand for information and less satisfaction with the information received [16].

## Discussion

This scoping review identified 56 studies reporting the HISB of people with IBD and provides an overview of characteristics and associated factors of HISB among patients with IBD in the digital era. Firstly, the content and sources of health information were clearly presented. Furthermore, we identified the types and factors of HISB among patients with IBD based on Wilson’s model of information behaviour. We anticipate that, to some extent, the framework of this paper can assist researchers in properly positioning their research aims in subsequent studies so that the objectives correspond to particular dimensions for in-depth empirical inquiry.

In our review, the results revealed diverse information needs among patients with IBD, encompassing fundamental aspects such as IBD-related information, treatment details, self-management strategies, and considerations related to sex and reproductive health, which align with previous reviews [19, 20, 89]. Furthermore, our analysis underscores the importance of prioritizing patients' information needs, given their dissatisfaction with current information and the range of requirements [19, 82]. In a recent scoping review, researchers emphasized nutritional information as a significant and frequently reported need among patients with IBD [89]. In contrast, the present scoping review demonstrated information about IBD treatment, especially medication information, was the most common and essential information to be met by patients in the included studies. This discrepancy may be because this review only included studies from 2010 onward, a period in which there have been significant changes and advances in pharmacotherapy as the primary treatment option for patients with IBD, such as the widespread adoption of biologics [3, 90]. Concerning sources of information, our review indicated that patients with IBD sought information through various channels. Face-to-face communication with HCPs, especially with IBD doctors and nurses, emerged as the preferred source due to its authority and credibility, consistent with findings in other reviews [20, 89]. However, the development of ICTs over the past decade has prompted an increasing number of patients to turn to online platforms, including social media and online communities, for supplementary information beyond what HCPs provide [14, 91, 92]. Despite the user-generated nature of ICTs being ideal for accessing a plethora of real-world experiences, concerns about the quality and reliability of the material may limit their utility [93, 94]. Some included studies stated patients often express concerns regarding the reliability and quality of information obtained through these channels [17, 60]. Furthermore, it is noteworthy that ICTs have reshaped the interactions of patients with healthcare providers. Some studies suggested that patients desired doctors to actively disseminate information on social platforms, which can facilitate easier access to high-quality information [60, 84]. This approach, which combines the strengths of both, may increasingly become the preferred method for patients seeking information.

To better understand patients' information-seeking behaviour, we used Wilson's model to analyse the types and factors of HISB among patients with IBD. Among patients with IBD, active searching appeared to be the predominant type of HISB, which is considered the most prevalent information-seeking behaviour in the Wilson’s Model [11]. Within our review, we noticed that social information seeking [95] is particularly visible. Because through social media, which is a key feature of Web 2.0 [96], patients can seek and share information with a broader range of people. Surprisingly, of all included studies, some showed ongoing searching behaviour in patients with IBD, similar to the findings in patients with other chronic diseases [97]. It may be because the characteristics of ICTs, such as diversity and accessibility, offer more possibilities for ongoing searches for people with IBD [15]. However, it is essential to note that passive seeking, including passive attention and passive searching, was identified in some included studies. Analogous observations in individuals with diabetes underscore the significance of attending to passive seeking due to its consequential impact on the medical decision-making process [98]. Notably, we also found information avoidance behaviour due to the negative information they perceived, which could lead to uninformed decisions and reinforcement of existing biases [99]. However, this phenomenon is rarely mentioned in the field of IBD, which requires further research to explore in depth.

The associated factors of HISB in patients with IBD, according to studies in the current scoping review, are echoed in the wider literature on health information seeking, especially about demographic characteristics [13, 100]. This review revealed that younger people utilized the Internet to find health information more frequently than older, and there was a significant connection between education level and active seeking behaviour. Besides, some included studies indicated that inactive information‐seekers were predominantly male. Source-related characteristics (e.g., credibility, usefulness) were also found to impact health information‐seeking. For instance, in a study exploring social media use among patients with IBD, Jason et al. identified significant concerns related to privacy/confidentiality and a lack of trust in posted information among patients with [81]. This finding was also reflected in a scoping review exploring the HISB of older adults, which indicated that the credibility and usefulness of sources could change the preferred information source [14]. In addition, as reflected in other reviews, the current review indicates factors related to disease and psychological aspects are part of the main associated factors of HISB in patients, such as disease duration, disease severity, and disease-related anxiety [14, 19, 98]. While we qualitatively identified various factors associated HISB in patients with IBD, the current depth of exploration primarily extends to demographic characteristics, leaving other aspects less thoroughly examined. Future research endeavors should aim to delve deeper into these unexplored dimensions.

### Implications for future research

In general, this scoping review identifies the need for more in-depth studies of HISB in patients with IBD. Firstly, as for research methods, prevailing cross-sectional approaches lack depth in understanding HISB among patients with IBD. Our review underscores the necessity for repeated surveys to explore evolving HISB trends and further unveil the evolution of information-seeking themes or sources, enriching our comprehension of HISB dynamics among patients with IBD. Meanwhile, prior mixed methods research predominantly relied on quantitative questionnaire analyses and qualitative focus groups or interviews. Exploring a broader spectrum of methodologies for this subject in the future, such as eye tracking [101], desktop tracking [102], or think-aloud protocols with evaluation immediately after a health-related search [103], would offer more objective and intricate data. These approaches facilitate the analysis of patients' attention distribution and behavioral patterns during information search and browsing. For example, eye movement capture allows inferences about the extent of attention patients allocate to specific information and their preferences in information presentation. Next, as can be seen from the evidence above, current research explored many factors associated with HISB among patients with IBD. Future studies can explore the causal relationship between specific factors and HISB through various approaches, such as longitudinal research. Thirdly, while we qualitatively analyzed five types of HISB in patients with IBD based on the Wilson’s model, few of the included studies directly examined specific patient-seeking actions, such as passive attention and ongoing searching. It's worth noting that, due to the passive approach to information seeking, when participants were asked how they sought information, they tended to assume that the question was asking about their active information seeking. Future research and HCPs are suggested to place greater emphasis on the specific seeking actions of individuals with IBD, particularly focusing on passive seeking and avoidance seeking, to enhance a more comprehensive understanding of patients’ HISB. Additionally, with the development of the mobile internet and the internet of things, an increasing number of IBD patients turn to the Internet for information-seeking and sharing their views online. Future research should delve deeper into online HISB and social information-seeking among patients with IBD. Moreover, it could be explored the relationship between HISB and online health-related content generation by patients with IBD. Finally, existing educational interventions may not well meet the information needs of people with IBD, especially among newly diagnosed patients [104]. Our reviews revealed the most preferred source of information is HCPs. In the digital health era, integrating resources, such as establishing an HCP-guided online community on social media, can enhance patient information-seeking. Furthermore, in the upcoming human-centred artificial intelligence era, personalized computer-based information resources could be developed, which could provide targeted support for people with IBD with different types of health information behaviours in various sociocultural settings.

### Limitations

There are several limitations in this scoping review. First, the HISB among patients with IBD before 2010 was not reviewed, because we want to underline the HISB of contemporary patients with IBD. Besides, we did not search the grey literature and excluded articles not in Chinese or English, and it was inevitable that some meaningful literature would be missed. Moreover, we excluded articles on transition and paediatrics because we believed that a different, more focused review was needed at this particular time. Moreover, we did not conduct an analysis of temporal variations in the literature. As a result, future research could explore the application of knowledge graphs to systematically delineate article themes across various stages. This methodological approach holds promise for uncovering the nuanced impact of evolving technological and socio-cultural changes on HISB in individuals with IBD over time [14, 105, 106]. What’s more, given the limited scope of our study, we did not include research directly analysing the association between HISB and health behaviours or outcomes (such as treatment adherence, disease remission, and quality of life) in patients. However, we acknowledge that exploring the relationship between HISB and health outcomes in patients with IBD is an important area for future research. Further, there are some inherent limitations of the Wilson's model [32]. It may not fully capture the nuanced HISB specific to individuals with IBD, and variations among individuals can exist. Moreover, the model's applicability depends on contextual factors and the unique characteristics of the studied population. To address these limitations as much as possible, our research employed a combination of inductive and deductive analyses, uncovering and exploring new categories beyond the scope of the Wilson’s model.

## Conclusions

This scoping review provides an overview of research on the health information-seeking behaviour of patients with IBD. It demonstrates the diverse information needs and sources among patients with IBD. Notably, medication information emerged as the most crucial information demand. While the internet was the most frequently mentioned source, direct consultation with HCPs was the most preferred source of information. Active searching, especially social information seeking, was observed to be the dominant information-seeking behaviour in the digital era for patients with IBD. Additionally, their information-seeking behaviours were influenced by a combination of diverse associated factors, such as resource-related and personal-related factors. However, this review highlights the need for further research on health information behaviour in the context of IBD, despite the increasing number of studies. Future research should focus on an in-depth exploration of the HISB among patients with IBD, including longitudinal changes. Moreover, efforts should be made to develop information resources that are both convenient and provide credible information services, although the development of such resources requires further investigation and evaluation.

### Supplementary Information

**Additional file 1.**

**Additional file 2.**

## Data Availability

The datasets used and/or analysed during the current study are available from the corresponding author on reasonable request.

## Abbreviations

CD: Crohn’s Disease
UC: Ulcerative Colitis
IBD: Inflammatory Bowel Disease
HISB: Health Information-Seeking Behaviour
CCCF: The China Crohn’s & Colitis Foundation
CCFA: Crohn's & Colitis Foundation
CDC: Centers for Disease Control and Prevention
ICTs: Information and Communication Technologies
HCPs: Healthcare Professionals

## References

[1] Kaplan GG, Windsor JW (2021). The four epidemiological stages in the global evolution of inflammatory bowel disease. Nat Rev Gastroenterol Hepatol.

[2] Ananthakrishnan AN, Kaplan GG, Bernstein CN, Burke KE, Lochhead PJ, Sasson AN (2022). Lifestyle, behaviour, and environmental modification for the management of patients with inflammatory bowel diseases: an International Organization for Study of Inflammatory Bowel Diseases consensus. Lancet Gastroenterol Hepatol.

[3] Na S-Y, Moon W (2019). Perspectives on Current and Novel Treatments for Inflammatory Bowel Disease. Gut Liver.

[4] Zhang Z, Yang H, He J, Lu X, Zhang R (2021). The Impact of Treatment-Related Internet Health Information Seeking on Patient Compliance. Telemed J E Health.

[5] Lim HM, Dunn AG, Lim JR, Abdullah A, Ng CJ (2022). Association between online health information-seeking and medication adherence: A systematic review and meta-analysis. Digit Health.

[6] McMullan RD, Berle D, Arnáez S, Starcevic V (2019). The relationships between health anxiety, online health information seeking, and cyberchondria: Systematic review and meta-analysis. J Affect Disord.

[7] Starcevic V, Berle D (2013). Cyberchondria: towards a better understanding of excessive health-related Internet use. Expert Rev Neurother.

[8] Luo A, Qin L, Yuan Y, Yang Z, Liu F, Huang P (2022). The Effect of Online Health Information Seeking on Physician-Patient Relationships: Systematic Review. J Med Internet Res.

[9] Chen Y-Y, Li C-M, Liang J-C, Tsai C-C (2018). Health Information Obtained From the Internet and Changes in Medical Decision Making: Questionnaire Development and Cross-Sectional Survey. J Med Internet Res.

[10] Gibson L, Tan ASL, Freres D, Lewis N, Martinez L, Hornik RC (2016). Nonmedical information seeking amid conflicting health information: negative and positive effects on prostate cancer screening. Health Commun.

[11] Wilson TD (2007). Looking for information: a survey of research on information seeking, needs, and behavior, 2nd edition. Inf Res.

[12] Lambert SD, Loiselle CG (2007). Health information seeking behavior. Qual Health Res.

[13] Zimmerman MS, Shaw G (2020). Health information seeking behaviour: a concept analysis. Health Info Libr J.

[14] Zhao YC, Zhao M, Song S (2022). Online Health Information Seeking Behaviors Among Older Adults: Systematic Scoping Review. J Med Internet Res.

[15] Wang X, Shi J, Kong H (2021). Online Health Information Seeking: A Review and Meta-Analysis. Health Commun.

[16] Pittet V, Vaucher C, Maillard MH, Girardin M, de Saussure P, Burnand B (2016). Information Needs and Concerns of Patients with Inflammatory Bowel Disease: What Can We Learn from Participants in a Bilingual Clinical Cohort?. PLoS ONE.

[17] Chamorro-de-Vega E, Romero-Jiménez R, Escudero-Vilaplana V, Ais-Larisgoitia A, Lobato Matilla ME, González CM (2022). Information and Communication Technologies in Patients With Immune-Mediated Inflammatory Diseases: Cross-sectional Survey. J Med Internet Res.

[18] Yu Q, Xu L, Li L, Zhi M, Gu Y, Wang X (2019). Internet and WeChat used by patients with Crohn’s disease in China: a multi-center questionnaire survey. BMC Gastroenterol.

[19] Al Khoury A, Balram B, Bessissow T, Afif W, Gonczi L, Abreu M (2022). Patient Perspectives and Expectations in Inflammatory Bowel Disease: A Systematic Review. Dig Dis Sci.

[20] Volpato E, Bosio C, Previtali E, Leone S, Armuzzi A, Pagnini F (2021). The evolution of IBD perceived engagement and care needs across the life-cycle: a scoping review. BMC Gastroenterol.

[21] Arksey  H,  O‘Malley L (2005). Scoping studies: towards a methodological framework. Int J Soc Res Methodol.

[22] Tricco AC, Lillie E, Zarin W, O’Brien KK, Colquhoun H, Levac D (2018). PRISMA Extension for Scoping Reviews (PRISMA-ScR): Checklist and Explanation. Ann Intern Med.

[23] Munn Z, Peters MDJ, Stern C, Tufanaru C, McArthur A, Aromataris E (2018). Systematic review or scoping review? Guidance for authors when choosing between a systematic or scoping review approach. BMC Med Res Methodol.

[24] Yoo Y, Boland RJ, Lyytinen K, Majchrzak A (2012). Organizing for innovation in the digitized world. Organ Sci.

[25] Yoo Y, Henfridsson O, Lyytinen K (2010). Research commentary—the new organizing logic of digital innovation: an agenda for information systems research. Inf Syst Res.

[26] Heaton-Shrestha C, Hanson K, Quirke-McFarlane S, Delaney N, Vandrevala T, Bearne L (2023). Exploring how members of the public access and use health research and information: a scoping review. BMC Public Health.

[27] Smailhodzic E, Hooijsma W, Boonstra A, Langley DJ (2016). Social media use in healthcare: A systematic review of effects on patients and on their relationship with healthcare professionals. BMC Health Serv Res.

[28] Sikich N NI. Quality Control Tool for Screening Titles and Abstracts by seco nd Reviewer: QCTSTAR. J Biom Biostat. 2015;06:230.

[29] Finfgeld-Connett D (2014). Use of content analysis to conduct knowledge-building and theory-generating qualitative systematic reviews. Qual Res.

[30] Wanderås MR, Abildsnes E, Thygesen E, Martinez SG (2023). Video consultation in general practice: a scoping review on use, experiences, and clinical decisions. BMC Health Serv Res.

[31] Vaismoradi M, Turunen H, Bondas T (2013). Content analysis and thematic analysis: Implications for conducting a qualitative descriptive study. Nurs Health Sci.

[32] Wilson TD (1997). Information behaviour: An interdisciplinary perspective. Inf Process Manage.

[33] Cavanagh S (1997). Content analysis: concepts, methods and applications. Nurse Res.

[34] Niedźwiedzka B (2003). A proposed general model of information behaviour. Inf Res.

[35] Bo J, Liu X, Jia J, Wang Y, Chen Y (2022). Self-management needs of patient with inflammatory bowel disease from perspective of informatization: a qualitative research. Military Nursing.

[36] Lin S, Chen Y, Cao Q (2018). Ways of acquiring and needs of requiring disease-related knowledge among patients with inflammatory bowel disease. Chin J Gen Pract.

[37] Gu J, Xiang Y, Yan S, Zhu L (2021). Analysis on the user behavior of utilizing the information support platform for inflammatory bowel disease. Military Nursing.

[38] Gu J, Lu X, Niu M, Zhu L (2016). Inflammatory bowel disease patient's experience of information seeking: a qualitative research. Chinese Nursing Management.

[39] Pan J, Bian Q, Wang X, Tu Y, Ding W, Xie L (2019). Investigation and analysis of the health education needs in patients with inflammatory bowel disease. Journal of Bengbu Medical College.

[40] Gui L, Ye Q, Wang M, Wang M, Zhang Q (2022). A qualitative study on health education needs of patients with inflammatory bowel disease. Practical Clinical Medicine.

[41] Sun S, Hu Y, Li H, Chen J, Lou Y, Weng C (2023). Patients’ perspectives on, experience with and concerns about crohn’s disease: insights from Chinese social media. BMC Gastroenterol.

[42] Britt RK (2017). Online Social Support for Participants of Crohn’s and Ulcerative Colitis Groups. Health Commun.

[43] Pérez-Pérez M, Pérez-Rodríguez G, Fdez-Riverola F, Lourenço A (2019). Using Twitter to Understand the Human Bowel Disease Community: Exploratory Analysis of Key Topics. J Med Internet Res.

[44] Yin R, Neyens DM (2020). Online Health Resource Use by Individuals With Inflammatory Bowel Disease: Analysis Using the National Health Interview Survey. J Med Internet Res.

[45] Keller R, Fusco S, Stange EF, Malek NP, Wehkamp J, Klag T. Infodemiology of Crohn’s disease and Ulcerative colitis using Google Trends - an approach to investigate patient needs. Zeitschrift fur Gastroenterologie. 2020;58:224–33. 10.1055/a-1068-2877 32018314

[46] Lee MJ, Jones GL, Lobo AJ, Brown SR (2001). pCD collaborators. Survey to define informational needs of patients undergoing surgery for Crohn’s anal fistula. Colorectal Dis.

[47] Lee MJ, Marshall JH, Jones GL, Lobo AJ, Brown SR. The informational and decisional preferences of patients undergoing surgery for Crohn’s anal fistula: a qualitative study. Colorectal Dis. 2020;22:703–12. 10.1111/codi.14936 31868981

[48] Wu Q, Zhong J (2018). Disease-related information requirements in patients with Crohn’s disease. Patient Prefer Adherence.

[49] Cohan JN, Ozanne EM, Hofer RK, Kelly YM, Kata A, Larsen C (2021). Ileostomy or ileal pouch-anal anastomosis for ulcerative colitis: patient participation and decisional needs. BMC Gastroenterol.

[50] Baker DM, Lee MJ, Jones GL, Brown SR, Lobo AJ (2017). The Informational Needs and Preferences of Patients Considering Surgery for Ulcerative Colitis: Results of a Qualitative Study. Inflamm Bowel Dis.

[51] Pittet V, Rogler G, Mottet C, Froehlich F, Michetti P, de Saussure P (2014). Patients’ information-seeking activity is associated with treatment compliance in inflammatory bowel disease patients. Scand J Gastroenterol.

[52] Bernstein KI, Promislow S, Carr R, Rawsthorne P, Walker JR, Bernstein CN (2011). Information needs and preferences of recently diagnosed patients with inflammatory bowel disease. Inflamm Bowel Dis.

[53] Aluzaite  K, Greveson  K, Ben-Horin  S, Leong R, Haj O, Schultz  M (2001). Barriers to international travel in inflammatory bowel disease patients. J Travel Med.

[54] van Erp LW, Neijenhuis MK, Heida W, Derwig J, Geleijns CE, Groenen MJM (2022). Improving Care for Recently Diagnosed Inflammatory Bowel Disease Patients: Lessons Learned From a Patient-Centred. Mixed-Method Study J Crohns Colitis.

[55] Goren I, Sharvit G, Godny L, Fatal SE, Barkan R, Hag O (2022). Exploring Popular Social Media Networks for Patients With Inflammatory Bowel Diseases: Insights for Better Care. J Clin Gastroenterol.

[56] Dos Santos Marques IC, Herbey II, Theiss LM, Shao CC, Fouad MN, Scarinci IC (2022). Understanding the surgical experience for Black and White patients with inflammatory bowel disease (IBD): The importance of health literacy. The American Journal of Surgery.

[57] Spinelli A, Carvello M, Adamina M, Panis Y, Warusavitarne J, Tulchinsky H (2021). Patients’ perceptions of surgery for inflammatory bowel disease. Colorectal Dis.

[58] Long  MD, Grewe  ME, Cerciello  E, Weisbein  L, Catabay  K, Kappelman  MD (2021). A Patient-Prioritized Agenda for Information Needs During the COVID-19 Pandemic: A Qualitative Study of Patients With Inflammatory Bowel Disease. Crohns Colitis 360.

[59] Cury DB, Paez LEF, Micheletti AC, Reis ST (2021). The Impact of Electronic Media on Patients with Inflammatory Bowel Disease. Risk Manag Healthc Policy.

[60] Chowdhary TS, Thompson J, Gayam S (2021). Social Media Use for Inflammatory Bowel Disease in a Rural Appalachian Population. Telemed J E Health.

[61] Aboubakr A, Riggs AR, Jimenez D, Mella MT, Dubinsky MC (2021). Identifying Patient Priorities for Preconception and Pregnancy Counseling in IBD. Dig Dis Sci.

[62] Khalil C, Van Deen W, Dupuy T, Bonthala N, Almario C, Spiegel B. Developing Patient-Centered Inflammatory Bowel Disease-Related Educational Videos Optimized for Social Media: Qualitative Research Study. JMIR medical education. 2020;6:e21639. 10.2196/21639 PMC7609199 33079065

[63] Zigron S, Bronstein J (2019). “Help is where you find it”: The role of weak ties networks as sources of information and support in virtual health communities. J Am Soc Inf Sci.

[64] Włodarczyk M, Włodarczyk J, Zalewska K, Olczyk M, Maryńczak K, Gajewski P (2019). Preferences of patients with inflammatory bowel disease for receiving specialized health services using technology: the role of Internet and other sources of medical information. Pol Przegl Chir.

[65] Marrie RA, Walker JR, Graff LA, Patten SB, Bolton JM, Marriott JJ (2019). Gender differences in information needs and preferences regarding depression among individuals with multiple sclerosis, inflammatory bowel disease and rheumatoid arthritis. Patient Educ Couns.

[66] Daher S, Khoury T, Benson A, Walker JR, Hammerman O, Kedem R (2019). Inflammatory bowel disease patient profiles are related to specific information needs: A nationwide survey. World J Gastroenterol.

[67] Philip V, Soubieres A, Poullis A (2018). Health concerns associated with travelling with inflammatory bowel disease (IBD): a questionnaire survey. Clin Med (Lond).

[68] McDermott E, Healy G, Mullen G, Keegan D, Byrne K, Guerandel A (2018). Patient Education in Inflammatory Bowel Disease: A Patient-Centred. Mixed Methodology Study J Crohns Colitis.

[69] Martín Fernández C, Maroto Martín C, Fernández SL (2018). Using the internet to evaluate the opinion of patients with inflammatory bowel disease with regard to the available information. Rev Esp Enferm Dig.

[70] Dibley L, Czuber-Dochan W, Wade T, Duncan J, Burch J, Warusavitarne J (2018). Patient Decision-Making About Emergency and Planned Stoma Surgery for IBD: A Qualitative Exploration of Patient and Clinician Perspectives. Inflamm Bowel Dis.

[71] Larsson K, Lööf L, Nordin K (2017). Stress, coping and support needs of patients with ulcerative colitis or Crohn’s disease: a qualitative descriptive study. J Clin Nurs.

[72] Yoo Y-S, Cho O-H, Cha K-S (2015). Disease-Related Knowledge and Information Needs Among Inflammatory Bowel Disease Patients in Korea. Gastroenterol Nurs.

[73] Catalán-Serra I, Huguet-Malavés JM, Mínguez M, Torrella E, Paredes JM, Vázquez N (2015). Information resources used by patients with inflammatory bowel disease: Satisfaction, expectations and information gaps. Gastroenterol Hepatol.

[74] Becker HM, Grigat D, Ghosh S, Kaplan GG, Dieleman L, Wine E (2015). Living with inflammatory bowel disease: A Crohn’s and Colitis Canada survey. Can J Gastroenterol Hepatol.

[75] Lesnovska KP, Börjeson S, Hjortswang H, Frisman GH (2014). What do patients need to know? Living with inflammatory bowel disease. J Clin Nurs.

[76] Viazis N, Mantzaris G, Karmiris K, Polymeros D, Kouklakis G, Maris T (2013). Inflammatory bowel disease: Greek patients’ perspective on quality of life, information on the disease, work productivity and family support. Ann Gastroenterol.

[77] Wong S, Walker JR, Carr R, Graff LA, Clara I, Promislow S (2012). The information needs and preferences of persons with longstanding inflammatory bowel disease. Can J Gastroenterol.

[78] Echarri A, Pérez-Calle JL, Calvo M, Molina G, Sierra-Ausín M, Morete-Pérez MC (2022). Should Inflammatory Bowel Disease Clinicians Provide Their Patients with e-Health Resources? Patients’ and Professionals’ Perspectives. Telemedicine and e-Health.

[79] Grunert PC, Reuken PA, Stallhofer J, Teich N, Stallmach A. Inflammatory Bowel Disease in the COVID-19 Pandemic: the Patients’ Perspective. Journal of Crohn’s & colitis. 2020;14:1702–8. 10.1093/ecco-jcc/jjaa126 PMC7337669 32564068

[80] Anker AE, Reinhart AM, Feeley TH (2011). Health information seeking: a review of measures and methods. Patient Educ Couns.

[81] Reich J, Guo L, Groshek J, Weinberg J, Chen W, Martin C, et al. Social Media Use and Preferences in Patients With Inflammatory Bowel Disease. Inflammatory bowel diseases. 2019;25:587–91. 10.1093/ibd/izy280 30203036

[82] Karadag P, Morris B, Woolfall K (2022). The information and support needs of patients living with inflammatory bowel disease: A qualitative study. Chronic Illn.

[83] Nachury M, Bouhnik Y, Serrero M, Filippi J, Roblin X, Kirchgesner J (2021). Patients’ real-world experience with inflammatory bowel disease: A cross-sectional survey in tertiary care centres from the GETAID group. Dig Liver Dis.

[84] Reich J, Guo L, Hall J, Tran A, Weinberg J, Groshek J (2016). A Survey of Social Media Use and Preferences in Patients with Inflammatory Bowel Disease. Inflamm Bowel Dis.

[85] Burisch J, Vegh Z, Pedersen N, Cuković-Čavka S, Turk N, Kaimakliotis I (2014). Health care and patients’ education in a European inflammatory bowel disease inception cohort: an ECCO-EpiCom study. J Crohns Colitis.

[86] Blumenstein I, McDermott E, Keegan D, Byrne K, Ellison M, Doherty G (2013). Sources of information and factual knowledge in Europeans with inflammatory bowel diseases: a cross-cultural comparison between German and Irish patients. J Crohns Colitis.

[87] Cullen G, Donnellan F, Long S, Forry M, Murray FE (2010). Perceptions of medication safety among patients with inflammatory bowel disease. Scand J Gastroenterol.

[88] Selinger CP, Carbery I, Warren V, Rehman AF, Williams CJ, Mumtaz S (2017). The relationship between different information sources and disease-related patient knowledge and anxiety in patients with inflammatory bowel disease. Aliment Pharmacol Ther.

[89] Norouzkhani N, Faramarzi M, Ghodousi Moghadam S, Karimi MA, Shokri Shirvani J, Bahari A (2023). Identification of the informational and supportive needs of patients diagnosed with inflammatory bowel disease: a scoping review. Front Psychol.

[90] Luo H, Cao G, Luo C, Tan D, Vong CT, Xu Y (2022). Emerging pharmacotherapy for inflammatory bowel diseases. Pharmacol Res.

[91] Finney Rutten LJ, Blake KD, Greenberg-Worisek AJ, Allen SV, Moser RP, Hesse BW (2019). Online Health Information Seeking Among US Adults: Measuring Progress Toward a Healthy People 2020 Objective. Public Health Rep.

[92] Chi Y, He D, Jeng W (2020). Laypeople’s source selection in online health information-seeking process. J Am Soc Inf Sci.

[93] Lee K, Hoti K, Hughes JD, Emmerton L (2014). Dr Google and the consumer: a qualitative study exploring the navigational needs and online health information-seeking behaviors of consumers with chronic health conditions. J Med Internet Res.

[94] Benetoli A, Chen TF, Aslani P (2019). Consumer perceptions of using social media for health purposes: Benefits and drawbacks. Health Informatics J.

[95] Shah C (2017). Social Information Seeking.

[96] Sanz-Lorente  M, Wanden-Berghe  C, Castejón-Bolea  R, Sanz-Valero  J (2018). Web 2.0 Tools in the Prevention of Curable Sexually Transmitted Diseases: Scoping Review. J Med Internet Res.

[97] Soong A, Au ST, Kyaw BM, Theng YL, Tudor CL (2020). Information needs and information seeking behaviour of people with dementia and their non-professional caregivers: a scoping review. BMC Geriatr.

[98] Kuske S, Schiereck T, Grobosch S, Paduch A, Droste S, Halbach S (2017). Diabetes-related information-seeking behaviour: a systematic review. Syst Rev.

[99] Link E (2021). Information avoidance during health crises: Predictors of avoiding information about the COVID-19 pandemic among german news consumers. Inf Process Manag.

[100] Mirzaei A, Aslani P, Luca EJ, Schneider CR (2021). Predictors of Health Information-Seeking Behavior: Systematic Literature Review and Network Analysis. J Med Internet Res.

[101] Kessler SH, Zillich AF (2019). Searching Online for Information About Vaccination: Assessing the Influence of User-Specific Cognitive Factors Using Eye-Tracking. Health Commun.

[102] Urman A, Makhortykh M (2023). You are how (and where) you search? Comparative analysis of web search behavior using web tracking data. J Comput Soc Sci.

[103] Macias W, Lee M, Cunningham N (2018). Inside the Mind of the Online Health Information Searcher using Think-Aloud Protocol. Health Commun.

[104] Navarro-Correal E, Ibarz A, Basagaña-Farres M, Feijoo-Cid M, Espart A, Selva L (2023). Educational Interventions for Newly Diagnosed Patients With Inflammatory Bowel Disease: A Scoping Review. Gastroenterol Nurs.

[105] Ji S, Pan S, Cambria E, Marttinen P, Yu PS (2022). A Survey on Knowledge Graphs: Representation, Acquisition, and Applications. IEEE Trans Neural Netw Learn Syst.

[106] Gao L, Yang T, Xue Z, Chan CKD (2023). Hot Spots and Trends in the Relationship between Cancer and Obesity: A Systematic Review and Knowledge Graph Analysis. Life (Basel).

