# Supplementary material for: Characteristics and associated factors of health information-seeking behaviour among patients with inflammatory bowel disease in the digital era: a scoping review
Source: BMC Public Health. 2024 Jan 27;24:307. doi: 10.1186/s12889-024-17758-w (PMC10821566; doi:10.1186/s12889-024-17758-w)
Supplement: Supplementary file 1 — Additional file 1. [file 12889_2024_17758_MOESM1_ESM.doc]

**Characteristics and associated factors of health information-seeking behavior among patients with inflammatory bowel disease in the digital era: a scoping review**

**Supplementary Material 1**

**Table 1: Search strategy**

From 2010 to April 10, 2023

| **Database** | **Search Terms** | **Result** |
| --- | --- | --- |
| **Pub****Med:** | **#9** ((("Inflammatory Bowel Diseases"[Mesh] OR "Crohn Disease"[Mesh] OR "Colitis, Ulcerative"[Mesh]) OR (inflammatory bowel disease[Title/Abstract] OR Crohn disease[Title/Abstract] OR ulcerative colitis[Title/Abstract] OR IBD[Title/Abstract] OR CD[Title/Abstract] OR UC[Title/Abstract])) AND (("Information Seeking Behavior"[Mesh] OR "Information Sources"[Mesh] OR "Help-Seeking Behavior"[Mesh] OR "Consumer Health Information"[Mesh] OR "Patient Education as Topic"[Mesh] OR "Health Communication"[Mesh]) OR (Information Seek*[Title/Abstract] OR information-seeking behavio*[Title/Abstract] OR Help Seek*[Title/Abstract] OR help-seeking behavio*[Title/Abstract] OR Information Source*[Title/Abstract] OR information access*OR information requirement*[Title/Abstract] OR information support*[Title/Abstract] OR information preference*[Title/Abstract] OR information seeking preference*[Title/Abstract] OR health information seeking behavio*[Title/Abstract] OR Health Communication*[Title/Abstract] OR information need*[Title/Abstract] OR Patient Education as Topic[Title/Abstract] OR ISB[Title/Abstract] OR HISB[Title/Abstract]))) AND (patient[MeSH Terms] OR patient[Title/Abstract]) from 2010 - 2023 581  **#8** ((("Inflammatory Bowel Diseases"[Mesh] OR "Crohn Disease"[Mesh] OR "Colitis, Ulcerative"[Mesh]) OR (inflammatory bowel disease[Title/Abstract] OR Crohn disease[Title/Abstract] OR ulcerative colitis[Title/Abstract] OR IBD[Title/Abstract] OR CD[Title/Abstract] OR UC[Title/Abstract])) AND (("Information Seeking Behavior"[Mesh] OR "Information Sources"[Mesh] OR "Help-Seeking Behavior"[Mesh] OR "Consumer Health Information"[Mesh] OR "Patient Education as Topic"[Mesh] OR "Health Communication"[Mesh]) OR (Information Seek*[Title/Abstract] OR information-seeking behavio*[Title/Abstract] OR Help Seek*[Title/Abstract] OR help-seeking behavio*[Title/Abstract] OR Information Source*[Title/Abstract] OR information access*OR information requirement*[Title/Abstract] OR information support*[Title/Abstract] OR information preference*[Title/Abstract] OR information seeking preference*[Title/Abstract] OR health information seeking behavio*[Title/Abstract] OR Health Communication*[Title/Abstract] OR information need*[Title/Abstract] OR Patient Education as Topic[Title/Abstract] OR ISB[Title/Abstract] OR HISB[Title/Abstract]))) AND (patient[MeSH Terms] OR patient[Title/Abstract]) 978  **#7** patient[MeSH Terms] OR patient[Title/Abstract] 2,872,430  **#6** ("Information Seeking Behavior"[Mesh] OR "Information Sources"[Mesh] OR "Help-Seeking Behavior"[Mesh] OR "Consumer Health Information"[Mesh] OR "Patient Education as Topic"[Mesh] OR "Health Communication"[Mesh]) OR (Information Seek*[Title/Abstract] OR information-seeking behavio*[Title/Abstract] OR Help Seek*[Title/Abstract] OR help-seeking behavio*[Title/Abstract] OR Information Source*[Title/Abstract] OR information access*OR information requirement*[Title/Abstract] OR information support*[Title/Abstract] OR information preference*[Title/Abstract] OR information seeking preference*[Title/Abstract] OR health information seeking behavio*[Title/Abstract] OR Health Communication*[Title/Abstract] OR information need*[Title/Abstract] OR Patient Education as Topic[Title/Abstract] OR ISB[Title/Abstract] OR HISB[Title/Abstract]) 1,482,678  #5 Information Seek*[Title/Abstract] OR information-seeking behavio*[Title/Abstract] OR Help Seek*[Title/Abstract] OR help-seeking behavio*[Title/Abstract] OR Information Source*[Title/Abstract] OR information access*OR information requirement*[Title/Abstract] OR information support*[Title/Abstract] OR information preference*[Title/Abstract] OR information seeking preference*[Title/Abstract] OR health information seeking behavio*[Title/Abstract] OR Health Communication*[Title/Abstract] OR information need*[Title/Abstract] OR Patient Education as Topic[Title/Abstract] OR ISB[Title/Abstract] OR HISB[Title/Abstract] 34,618  **#4** "Information Seeking Behavior"[Mesh] OR "Information Sources"[Mesh] OR "Help-Seeking Behavior"[Mesh] OR "Consumer Health Information"[Mesh] OR "Patient Education as Topic"[Mesh] OR "Health Communication"[Mesh]  1,457,579  **#3** ("Inflammatory Bowel Diseases"[Mesh] OR "Crohn Disease"[Mesh] OR "Colitis, Ulcerative"[Mesh]) OR (inflammatory bowel disease[Title/Abstract] OR Crohn disease[Title/Abstract] OR ulcerative colitis[Title/Abstract] OR IBD[Title/Abstract] OR CD[Title/Abstract] OR UC[Title/Abstract]) 285,250  **#2** inflammatory bowel disease[Title/Abstract] OR Crohn disease[Title/Abstract] OR ulcerative colitis[Title/Abstract] OR IBD[Title/Abstract] OR CD[Title/Abstract] OR UC[Title/Abstract] 256,294  **#1** "Inflammatory Bowel Diseases"[Mesh] OR "Crohn Disease"[Mesh] OR "Colitis, Ulcerative"[Mesh] 95,687 | **581** |
| **Web of Science** | **#1** TS=(ulcerative colitis OR Inflammatory Bowel Diseases OR Crohn Disease OR CD OR UC) 920326  **#2** TS=(information-seeking behavio* OR Information Source* OR information requirement* OR information support* OR information preference* OR information need* OR Patient Education as Topic OR ISB OR HISB) 2706834  **#3** TS=(patient) 13046894  **#4** #1 AND #2 AND #3 and Preprint (exclude - database) and 2010 or 2011 or 2012 or 2013 or 2014 or 2015 or 2016 or 2017 or 2018 or 2019 or 2020 or 2021 or 2022 or 2023 2669 | **2669** |
| **EMBASE** | **#15** #13 AND #14 AND ([embase]/lim OR [medline]/lim) AND [2010-2023]/py 835  **#14** 'patient':ti,ab,kw 4,347,463  **#13**  #3 AND #12 2,093  **#12** #10 OR #11 261,081  **#11** 'information seek*':ti,ab,kw OR 'information-seeking behavio*':ti,ab,kw OR 'help seek*':ti,ab,kw OR 'help-seeking behavio*':ti,ab,kw OR 'information source*':ti,ab,kw OR 'information access*':ti,ab,kw OR 'information requirement*':ti,ab,kw OR 'information support*':ti,ab,kw OR 'information preference*':ti,ab,kw OR 'information seeking preference*':ti,ab,kw OR 'health information seeking behavio*':ti,ab,kw OR 'health communication*':ti,ab,kw OR 'information need*':ti,ab,kw OR 'patient education as topic':ti,ab,kw OR 'isb':ti,ab,kw OR 'hisb':ti,ab,kw 45,250  **#10** #4 OR #5 OR #6 OR #7 OR #8 OR #9 229,195  **#9** 'medical information'/exp 85,938  **#8** 'patient education'/exp 123,637  **#7** 'consumer health information'/exp 4,244  **#6** 'help seeking behavior'/exp 15,362  **#5** 'information source'/exp 1,151  **#4** 'information seeking'/exp 5,539  **#3** #1 OR #2 438,740  **#2** 'inflammatory bowel disease':ti,ab,kw OR 'crohn disease':ti,ab,kw OR 'ulcerative colitis':ti,ab,kw OR 'ibd':ti,ab,kw OR 'cd':ti,ab,kw OR 'uc':ti,ab,kw 377,049  **#1** 'inflammatory bowel disease'/exp 197375 | **835** |
| **CINAHL** | **S4** ( S1 AND S2 ) AND AB patient 138  **S3** S1 AND S2 293  **S2** SU ( Information Seeking Behavior OR Information Sources OR Help-Seeking Behavior OR Consumer Health Information OR Patient Education as Topic OR Health Communication ) OR AB ( Information Seek* OR information-seeking behavio* OR Help Seek* OR help-seeking behavio* OR Information Source* OR information access*OR information requirement* OR information support* OR information preference* OR information seeking preference* OR health information seeking behavio* OR Health Communication* OR information need* OR Patient Education as Topic OR ISB OR HISB ) 82,484  **S1** SU ( inflammatory bowel disease OR Crohn disease OR ulcerative colitis ) OR AB ( inflammatory bowel disease OR Crohn disease OR ulcerative colitis OR IBD OR CD OR UC ) 35,252 | **138** |
| **PsycINFO** | **S5** S3 AND S4 databases - APA PsycInfo;APA PsycArticles;Psychology and Behavioral Sciences Collection 95  **S4** AB patient Conditions - Date of publication: 20100101-20230431 475,459  **S3** S1 AND S2 Conditions - Date of publication: 20100101-20230431 199  **S2** SU ( Information Seeking Behavior or Information Sources or Help-Seeking Behavior OR Consumer Health Information or patient education as topic or health communication ) OR ( Information Seek* OR information-seeking behavio* OR Help Seek* OR help-seeking behavio* OR Information Source* OR information access*OR information requirement* OR information support* OR information preference* OR information seeking preference* OR health information seeking behavio* OR Health Communication* OR information need* OR Patient Education as Topic OR ISB OR HISB ) 116,583  **S1** SU ( inflammatory bowel disease or crohn's disease or ulcerative colitis ) OR ( inflammatory bowel disease or crohn's disease or ulcerative colitis or ibd or cd or uc ) 18,992 | **95** |
| **China National Knowledge Infrastructure (CNKI)** | TKA='炎症性肠病'+'克罗恩病'+'溃疡性结肠炎'+'IBD'+'CD'+'UC' AND TKA='信息寻求'+'信息需求'+'信息获取'+'信息来源'+'信息偏好'+'信息搜索'+'ISB'+'HISB' + '信息支持' AND TKA='患者'  ***English-translated version:***  TKA='Inflammatory Bowel Disease' + 'Crohn's Disease' + 'Ulcerative Colitis' + 'IBD' + 'CD' + 'UC' AND TKA='Information Seeking' + 'Information Needs' + 'Information Acquisition' + 'Information Sources' + 'Information Preferences' + 'Information Searches' + 'ISB' + 'HISB' + 'Information Support' AND TKA='Patients' | **45** |
| **China Wanfang Database** | (题名或关键词:(炎症性肠病 OR 克罗恩病 OR 溃疡性结肠炎 OR IBD OR CD OR UC ) and 题名或关键词:(信息需求 OR 信息获取 OR 信息寻求 OR 信息偏好 OR 信息搜索 OR 信息来源 OR ISB OR HISB OR 信息支持)) and Date:2010-*  ***English-translated version:***  (Title or Keywords: (Inflammatory Bowel Disease OR Crohn's Disease OR Ulcerative Colitis OR IBD OR CD OR UC ) AND Title or Keywords: (Information Needs OR Information Acquisition OR Information Seeking OR Information Preferences OR Information Searches OR Information Sources OR ISB OR HISB OR Information Support)) AND Date:2010-* | **18** |
| **China Biology Medicine Disc (CBMdisc)** | 1) (( "炎症性肠病"[加权:扩展] OR "克罗恩病"[加权:扩展] OR "溃疡性结肠炎"[加权:扩展]) OR( "IBD"[常用字段:智能] OR "UC"[常用字段:智能] OR "CD"[常用字段:智能])) AND 2010-2023[日期] 121773  2) ("信息寻求行为"[加权:扩展] OR( "信息需求"[常用字段:智能] OR "信息获取"[常用字段:智能] OR "信息寻求"[常用字段:智能] OR "信息偏好"[常用字段:智能] OR "信息搜索"[常用字段:智能] OR "信息来源"[常用字段:智能] OR "ISB"[常用字段:智能] OR "HISB"[常用字段:智能] OR "信息支持"[常用字段:智能])) AND 2010-2023[日期] 6469  3) (#2) AND (#1) 24  ***English-translated version:***  1) (("Inflammatory Bowel Disease" [weighted: extended] OR "Crohn's Disease" [weighted: extended] OR "Ulcerative Colitis" [weighted: extended]) OR ("IBD" [commonly used field: smart] OR "UC" [commonly used field: smart] OR "CD" [commonly used field: smart])) AND 2010-2023 [DATE] 121773  2) ("Information Seeking Behaviour" [weighted: extended] OR ("Information Needs" [common field: smart] OR "Information Access" [common field: smart] OR "Information Seeking" [common field: smart] OR "Information Preferences" [common field: smart] OR "Information Searches" [common field: smart] OR "Information Sources" [common field: smart] OR "ISB" [ Common Fields: smart] OR "HISB"[Common Fields: smart] OR "Information Support"[Common Fields: smart])) AND 2010-2023[Date] 6469  3) (#2) AND (#1) 24 | **24** |
