# Supplementary material for: Characteristics and associated factors of health information-seeking behaviour among patients with inflammatory bowel disease in the digital era: a scoping review
Source: BMC Public Health. 2024 Jan 27;24:307. doi: 10.1186/s12889-024-17758-w (PMC10821566; doi:10.1186/s12889-024-17758-w)
Supplement: Supplementary file 2 — Additional file 2. [file 12889_2024_17758_MOESM2_ESM.docx]

**Characteristics and associated factors of health information-seeking behavior among patients with inflammatory bowel disease in the digital era: a scoping review**

**Supplementary Material 2**

**Table1: Overview of included studies**

| **Author (Year/Country)** | **Study Design** | **Study Objective** | **Participants**  **(n)** | **Gender Male**  **/Female**  **(n)** | **Age (year)** | **Education Level (%)** | **Disease duration** | **Disease activity** |
| --- | --- | --- | --- | --- | --- | --- | --- | --- |
| Pan J el ta.  (2019/China)[1] | Cross sectional (an online questionnaire survey) | To understand the needs of the content，form and frequency of health education in patients with inflammatory bowel disease( IBD), and provide the evidence for effective health education activities for IBD patients． | UC:52, CD:132 | 107/77 | / | Primary or Junior High School:4.89%, High school or junior college:48.91%, Above junior college:46.20% | / | / |
| Ling S el ta.  (2018/China)[2] | Cross sectional (a questionnaire survey) | To survey the ways of acquiring and the needs of requiring disease-related knowledge among patients with inflammatory bowel disease (IBD). | CD:199, UC:160 | 226/133 | ＜30 year:82, 30-39 year:110, 40-49 year:57, 50-59 year:53, 60-69 year:41, ≥70 year:16 | Primary School:9.2%, Junior High School:25.1%, High school or Secondary Schools:24.8%, Junior college:17.0%, Bachelor degree:19.2%, Above bachelor degree:4.7% | / | / |
| Gui L el ta.  (2022/China)[3] | Qualitative (phenomenology, face-to-face semi-structured interview) | To explore the needs and suggeations of patients with inflammatory bowel disease(IBD) for health education. | CD:5, UC:8 | 8/5 | Range:19-61 | Junior High School:7.7%, High school:23.1%, Junior college:38.5%, Bachelor degree:30.7% | 1-23 years | / |
| Gu J el ta.  (2021/China)[4] | Cross sectional (questionnaire, analysis of information support platform data) | To explore the web browsing behavioural characteristics of users of information-supporting web platforms for inflammatory bowel disease. | CD:164, UC:74 | 145/93 | Mean:33.75, SD:9.31 | / | 1-3.75years | / |
| Gu J el ta.  (2016/China)[5] | Qualitative (phenomenology, face-to-face semi-structured interviews) | To understand inflammatory bowel disease(IBD) patient's information needs and their experience of information seeking in order to gather evidence to design information support program in future. | CD:12, UC:8 | 13/7 | 18-20:2, 21-30:5, 31-40:8, 40-50:3, ＞50:2 | Primary School:10.0%, Junior High School:20.0%, High school or Secondary Schools: 20.0%, Junior college or bachelor degree: 40.0%, Above bachelor degree:10.0% | ＜2 years:8, 2-5 years:5, 5-10years:4, ＞10years:3 | / |
| Bo J el ta.  (2022/China)[6] | Qualitative (descriptive research method, semi-structured in-depth interviews) | To understand the needs of patients with inflammatory bowel disease(IBD) for self-management from the perspective of informatization, and further promote the construction of informatization self-management mode. | CD:9, UC:4 | 9/4 | Mean:37.62 Range:19-73 | Junior High School: 15.4%, High school or Secondary Schools: 30.8%, Junior college or bachelor degree:38.5%, Above bachelor degree:15.4% | 1-8years | Remission: 23.1% Active:  76.9% |
| Bernstein KI  el ta.  (2011/Canada)  [7] | Cross sectional (survey) | The aim of this study was to assess the information needs and experiences of patients who were recently diagnosed with inflammatory bowel disease (IBD). | CD:34, UC:38, IBD-type unspecified:2 | 35/39 | Mean:37.8, SD:14.9 | No postsecondary:39%, Trades/non-university certificate or diploma/ below bachelor degree: 30%, Bachelor degree:26%, Above bachelor degree:6%, | 3 months to 2 years | Active(MIBDI): 68% |
| Aluzaite K el ta.  (2021/UK)[8] | Cross sectional (a multi-center online survey) | We aimed to investigate inflammatory bowel diseases (IBD)-associated challenges to travel, information-seeking behaviour and associated factors. | CD:1104, UC:670, Indeterminate colitis:53, Other/do not know:51 | 440/1431, Other/prefer not to say:4 | 18–29:567, 30–49:903, 50–69:265, 70–89:17 | Below high school:12.4%, High school:21.5%, Undergrad:51.3%, Postgrad:14.8% | ≤1 year:5.2%, 1–5 years:25.7%, 6–10 years:19.8%,  10+years:49.3% | Percent time flare up in the last 6 months : Never: 22.8%  25%: 37.0% 50%: 17.0%  75%: 12.2% All the time :11.0% |
| Qiao Y el ta.  (2019/China)[9] | Cross sectional (a multi-center questionnaire survey) | This survey was performed to study the use of the internet and social media by CD patients in China, to compare the most popular WeChat CD public accounts and to analyse their impact of the internet and social media on the medication adherence of IBD patients. | CD:342 | 205/137 | Mean:41, Range:31-45 | Illiteracy:0.3%, Primary school:3%, High school or technical school:36%, College:59%, Postgraduate education:2% | / | / |
| Sun S el ta.  (2023/China)  [10] | Cross sectional (Analysis of forum posts) | The aim of this study was to explore the experience and perceptions of patients with Crohn’s disease in China. | / | / | / | / | / | / |
| van Erp LW  el ta.  (2022/Netherlands)[11] | Mixed-method (semi-structured interviews, questionnaires) | This study aimed to evaluate care for recently diagnosed IBD patients from the patient perspective and assess themes for improvement. Secondarily, we aimed to assess HRQoL, anxiety and depression of recently diagnosed IBD patients. | Interviews: CD:7, UC:13 Questionnaires: CD:29, UC:45,IBD-U:7 | Interviews:  10/10 Questionnaires:  41/40 | Interviews: Median:34 [IQR :26–49] Questionnaire: Median:37  [IQR: 25–58] | Questionnaires: Low:19%,  Middle:49%, High:32% | Interviews:5-14 months | Remission [HBI≤4, SCCAI≤2]: 76%  Active disease [HBI>4, SCCAI>2]: 24% |
| Karadag P el ta.  (2022/UK)[12] | Qualitative (phenomenological approach, telephone semi-structured interviews) | To explore patients’ experiences of living with inflammatory bowel disease (IBD) with a focus on their information and support needs. | CD:7, UC:8 | 6/9 | Mean: 28 , Range: 20–40 | / | / | / |
| Goren I el ta.  (2022/Israeli)  [13] | Mix methods (analysis of forum posts, thematic analysis, online survey) | The aim was to assess topics of interest and concerns among patients with inflammatory bowel diseases (IBD) who are active online. | 2623 published posts on the HCP-guided SM network Survery: CD:338, UC:196, | 229/305 | Median:38, [IQR: 28.7–51.0] | Elementary:1%, High school:30%, Above high school:9%, Bachelor of Arts:31%, Master of Arts:22%, Doctor of Philosophy:2%, Professional training:4% | Mean:10 years,  [IQR:4-19] |  |
| Dos Santos Marques IC  el ta.  (2021/USA)[14] | Qualitative (Focus groups, interviews) | To characterize the surgical experience for Black and White IBD patients using qualitative methods. | CD:18, UC:9 (2 Black focus groups and 4 White focus groups,10 Black and 17 White individuals) | 14/13 | Mean :44.8  SD:13.2 | / | / | / |
| Chamorro-de-Vega E el ta.  (2022/Spain)  [15] | Cross sectional (a questionnaire survey) | The aim of this study is to describe the degree of use of ICTs in patients with IMIDs (including rheumatic diseases, inflammatory bowel diseases, and psoriasis), identify their needs, and analyze their interest in the use of apps as tools for better management of their disease. | CD:133, Rheumatoid arthritis:103, Spondyloarthritis:79, Psoriatic arthritis:62, Psoriasis:58,  UC:53, Other:48, More than one IMID:57 | 221/251 | ≤35:74 , 36-50:159, 51-60:128, ≥61:106, No response:5 | No education or incomplete primary education:2.1%, Primary education:16.9%, Secondary education:35.4%, University education:44.7%, No response:0.8% | / | / |
| Spinelli A el ta.  (2021/Italy)  [16] | Cross sectional (an international multilingual online survey) | The aim of this work was to investigate patients’ perceptions of surgery and the impact on reported outcomes. | CD :259,  UC: 153,  other IBD diagnoses:13 | 136/289 | Mean:38, SD:12 | / | / | / |
| Nachury M el ta.  (2021/France)  [17] | Cross sectional (survey) | To assess inflammatory bowel disease (IBD) patients’ experience with their disease, their treatment and their relationship with their physician. | CD:1363, UC:678 | 1067/944 | Median:40 [IQR: 29–52] | / | Median:10.5 [IQR:4.5-18.5] | Clinical remission: 9.1% |
| Long MD el ta.  (2021/USA)[18] | Qualitative (virtual focus group ) | We performed a qualitative study to better understand IBD patient experiences and concerns when navigating the COVID-19 pandemic, with the goal of prioritizing patients’ information needs. | IBD:31 (6 focus groups) | / | Range:18–99 | / | / | / |
| Pc G el ta.  (2020/Germany)[19] | Cross sectional (survey) | Our aims were to determine COVID-19 prevalence/exposure, perception and information sources, medication compliance, patient behaviour and physician contact among patients with IBD compared with non-IBD controls. | IBD:415, Non-IBD: 116 | 118/227 (IBD) 50/66 (non-IBD) | Mean: 45, Range: 18-82 (IBD) Mean: 48, Range: 18-82 (non-IBD) | Junior high school (Hauptschule) :10.3%, Secondary school (Real Schule):44.6%, High school: 13.8%, College degree: 31.3% | / | Remission :56.1%,  Mildly active: 30.6%  Chronically active: 12.0%  Flare :0.5% |
| Lee MJ el ta.  (2021/England)[20] | Cross sectional (a multi-center questionnaire survey) | The aim of this study was to identify what information patients would like in order to make treatment decisions and to explore experiences of making decisions in this setting. | CD:92 | 44/48 | Median :42, Range:19–87 | None:7.6%, GCSE:25.0%, A-level:23.9%, Bachelor's degree:25.0%, Higher degree, e.g., MSc/PhD:13.0%, Other:3.3% | Median :8 years , Range:0.08–37 years | / |
| Cury DB el ta.  (2020/Brazil)  [21] | Cross sectional (a questionnaire survey) | The aim of our study is to evaluate the use of the internet and its effects on patients with IBD. | CD:37, UC:8 | / | Mean: 41.3  SD:15.9 | / | / | / |
| Cohan JN. el ta.  (2021/USA)[22] | Qualitative (Semi-structured interviews) | We aimed to understand how patients decide between these two options. | UC:16 | 8/8 | Range:18–70 | High schoo:37.5%, College or more:56.3% Unknown:6.2% | / | / |
| Chowdhary TS el ta.  (2021/USA)[23] | Cross sectional (an online questionnaire survey) | The aim of this study was to evaluate social media use among patients with IBD in the state of West Virginia (WV). | IBD:624 | 422/202 | Mean:43.5 | High school:32.2%, Some college:23.1%, Bachelor's:25.8%, Master's plus:18.9% | / | / |
| Aboubakr A  el ta.  (2021/USA)[24] | Cross sectional (an online questionnaire survey) | Our study aimed to describe the priority rankings of topics selected by patients seeking preconception and pregnancy counseling. | CD:70, UC:43, IBD-U:3 | 0/116 | Median:32, [IQR:28-34] | / | / | CD remission (HBI < 4): 94%, UC remission (Partial Mayo ≤ 2): 73% |
| Yin R el ta.  (2020/USA)[25] | Cross sectional (2016 National Health Interview Survey weighted data) | Our goal was to investigate the factors that influence internet use in acquiring health information by individuals with inflammatory bowel diseases. Specifically, we identified factors associated with internet searching behavior and using the internet for completing health-related tasks. | IBD:3,155,477 | 1,123,455/2,032,022 | 18-35:454,950 36-55: 1,159,430 >55 :1,541,097 | / | / | / |
| Lee MJ el ta.  (2020/UK)[26] | Qualitative ( face-to-face semi-structured interviews) | The aim of this study was to investigate the informational and decisional preferences of patients when surgical treatment is being considered. | CD:17 | 9/8 | Median: 27  Range:19–71 | / | 6 months to 40 years | / |
| C K el ta.  (2020/USA)[27] | Qualitative (focus groups, face-to-face semi-structured interviews) | This study aims to gain an in-depth understanding of the unmet educational needs of patients with IBD and to use the resulting insights to develop a collection of freely available, evidence-based educational videos optimized for dissemination through social media. | IBD(Total):29 Phase1  CD:12, UC: 5 Phase2  CD:9, UC: 3 Phase3  CD:7, UC: 3 | Phase1 4/13 Phase2 4/8 Phase3 2/8 | Phase1  (focus group) Median:41 Range:22-83  (Interview) Median:34 Range:21-64 Phase2 Median:41 Range:23-83  Phase3 Median:25 Range:36-52 | Phase1  High school:17.6%,  College degree:41.2%,  Graduate degree:41.2% Phase2  College degree:50%,  Graduate degree:50% Phase3  High school:30%,  College degree:40%,  Graduate degree:30% | Phase1  (focus group) Median:15 Range:2-39  (interview) Median:12.5 Range:1-22 Phase2 Median:17.5 Range:2-39  Phase3 Median:19 Range:2-36 | / |
| R K el ta.  (2020/Germany)[28] | Cross sectional (analyzed the internet search behavior) | We aim to identify information gaps to allow a more complete education of patients. | / | / | / | / | / | / |
| Zigron S el ta.  (2019/Israel)  [29] | Qualitative  (In-depth interviews) | Hence, the purpose of the current study is to examine the activity of virtual health communities for users with a chronic disease that affects their daily lives (for instance, Crohn's), by understanding the role that these online spaces play as sources for information and social support and focusing on the nature of weak ties. | IBD:23 | 8/15 | Range:20-40 | / | / | / |
| Włodarczyk M el ta.  (2019/Poland)  [30] | Cross sectional (questionnaire) | The aim of the study was to characterize the extent of Internet use for health information among a representative sample of IBD patients and to examine the effects that Internet and other sources of information about specialized health services have on patients’ choices of a doctor and hospital department. | CD:91, UC:54 | 55/68 | (Women)  Mean:33.7 SD:12.8  (Men)  Mean:40.0 SD:15.5 | Primary:9.0%, Secondary:51.2%, Tertiary:39.8% | Mean: 5.1 SD: 6.4(Women) Mean: 5.6 SD: 5.9 (Men) | / |
| J R el ta.  (2019/USA)[31] | Cross-sectional  (survey) | The aim of this study was to assess social media use, patient preferences, and barriers to use in a large internet cohort of patients with IBD to target future appropriate educational interventions. | CD: 1271, UC/IC: 689 | 494/1466 | Median:42.5  [IQR:14.4] | ≤ high school :5.3% Some college :16.2% college graduate:44.1% graduate school:34.4% | / | Remission: 62.9% |
| Marrie R A el ta.  (2019/Canada)  [32] | Cross-sectional ( questionnaire) | To support future development of information resources we compared the information needs of persons with MS regarding depression with those of individuals with IBD or RA. We also compared information needs by gender. | IBD:114, MS:141, RA:73 | 83/245 (IBD:38/76) | Mean: 54.4, SD: 13.2 (IBD Mean:50.2, SD: 14.0) | <High school: 4.0% (IBD:3.5%) High school/ GED:22.6% (IBD: 24.6%)； >High school: 73.5% (IBD: 71.9%) | / | / |
| Daher S el ta.  (2019/Israel)  [33] | Cross-sectional (a 28-item questionnaire, a nationwide survey) | To evaluate the scope of disease-related knowledge among IBD patients and determine whether different patient profiles drive unique information needs. | CD:392, UC:179 | 265/240 | Mean:34.2 SD: 13.3 | High school completion:50%, Bachelor’s degree:33%,  Master’s degree :15%,  Doctorate:2% | / | Active (MIBDI): 69% |
| Wu Q el ta.  (2018/China)  [34] | Cross-sectional (questionnaire) | To explore the demands for different disease-related information and the acceptance of various information sources in patients with Crohn's disease (CD). | CD:159 | 114/45 | ≤25：61, 26-35：59, 36-45：21, ≥46：18 | Elementary ：3.8% Secondary：22.6% Higher：73.6% | ＜1：34.6%, 1-5：45.9% ＞31: 19.5% | / |
| Philip V el ta.  (2018/UK)[35] | Cross-sectional (a 32-question paper-based questionnaire) | The purpose of the study was to explore patient attitudes towards travelling and to identify areas of unmet need in the IBD service. | CD: 73, UC: 53,  unsure of their type of IBD: 7, indeterminate colitis: 3 | 70/66 | Mean: 40, Range:18-81 | / | Mean:12.2 years | / |
| McDermott E  el ta.  (2018/Ireland)  [36] | Mixed-methods (focus groups, questionnaire) | To assess patients’ education needs in IBD to facilitate design of a patient education programme. | focus groups CD:6, UC:6 questionnaire CD:184 UC:135 | focus groups: 6/6 questionnaire:159/163 | Questionnaire  Median:38, IQR:29-48 | Questionnaire No formal education :5%, Leaving certificate :42%, University :53% | Questionnaire Median:7 IQR:3-15 | / |
| Martín-Fernández C  el ta.  (2018/Spain)  [37] | Cross-sectional  (A 39-item survey) | The aims of the study were to ascertain how patients with inflammatory bowel disease (IBD) felt about the information available and the way that their doctors informed them. In addition, how patients used the internet and factors that predicted a positive information evaluation were also assessed. | CD:311 UC:105 U-IBD:4 | 126/294 | < 20 :5.2%, 20-40:49.8%, 40-60 :41.2%, > 60 :3.8% | No formal education:0.7%, Primary School: 13.3%, Secondary School:45.5%, University studies: 40.5% | < 5 years:32.6%, 5-10 years: 22.1%, 10-20 years:30.2% > 20 years :15% |  |
| Dibley L el ta.  (2018/UK)[38] | Qualitative ( focus groups, semi-structured interviews) | This qualitative study explored influences on patients' SFS decision-making and compared preoperative concerns with postoperative outcomes. | Focus groups CD: 5 , UC: 14  Patient interviews CD: 14, UC: 14, IBDU: 1 | Focus groups :9/10  Patient interviews :8/21 | Focus groups :  Range:20‒73, Mean:40.5  Patient interviews:  Range:22‒58, Mean: 39.4 | / | / | / |
| Selinger C P  el ta.  (2017/UK)[39] | Cross-sectional (questionnaire) | To determine the relationship between different information sources and patient knowledge and anxiety in patients with IBD. | CD:165, UC:142 | 121/186 | Mean:47.3  Range:17–88 | Secondary school:36.6%, Technical college:25.5%, University not  completed: 8.8%, University degree: 11.1%, Postgraduate degree:18% | / | Remission: CD (HBI):62.5%, UC (SCCAI):70.1% |
| Larsson K el ta.  (2017/Sweden)  [40] | Qualitative (telephone interviews or face-to-face interviews) | The aim of this study was to investigate the speciﬁc disease-related stress in individuals with UC or CD, how they cope with this stress and what help is requested from the health care. | CD:8, UC:7 | 6/9 | Mean:50, Range: 29–63 | / | ≤5 years:5,  >5 years:10 | / |
| Britt RK.  (2017/USA)[41] | Qualitative (analysis of forum posts) | The aim of our study was to explore the types of social support exchanges that emerged in a Crohn’s and ulcerative colitis online group. | / | / | / | / | / | / |
| Baker DM el ta.  (2017/UK)[42] | Qualitative (semi-structured interviews) | The aim of our study was to use established qualitative methods to describe patient informational needs and preferences when deciding between surgery and ongoing medical management for ulcerative colitis. | UC:16 | 7/9 | Median: 42, Range:22-74 | / | / | / |
| Reich J el ta.  (2016/USA)[43] | Cross-sectional (a 41-question survey) | The aim of this study was to assess social media usage and preferences in patients with IBD. | CD:89, UC:29 | 53/66 | Mean:40.6 | Grades 1 through 11 :7%, Grade12 or GED :26%, College 1-3 years: 28%, College 4 years or more: 39% | Mean:13.23 SD:12.18 | CD:HBI(mean): 3.4 UC: SSCAI(mean): 5.3 |
| Pittet V el ta.  (2016/Switzerland)[44] | Mixed-methods study (semi-narrative survey, focus groups) | To explore information expectations of patients included in a national bilingual IBD cohort in Switzerland (SIBDC). | CD:407, UC:321 | 346/382 | <35 years:217, 35–50 years:292, >50 years:219 | None or compulsory: 12.0%, Upper 2nd education:56.5%, Tertiary education:31.4% | <5 years: 234, 5–15 years: 281, >15 years: 201 | / |
| Yoo Y-S el ta.  (2015/Korea)  [45] | Cross-sectional (questionnaire) | The aim of this study was, first, to determine the difference in disease-related knowledge and information needs according to the general characteristics of IBD patients, and second, to compare the difference in knowledge and information needs of CD and UC patients. | CD:169, UC144 | 186/127 | 18-29:115, 30-39:101, 40-49:64, ≥50:33 | High school: 34.5%, ≥university education: 65.5% | <1 years:35, 1–5 years:83, 6–10 years:110, >10 years: 85 | / |
| Catalán-Serra I el ta.  (2015/Spain)  [46] | A multicenter, prospective, observational, cross-sectional cohort study (questionnaire survey) | The objective of the study was to determine the information resources these patients used, together with their perceived information gaps and expected preferences. | CD:216, UC:163 | 190/189 | Mean:37.9, SD:11.5, Range:16-76 | Tertiary:24%, Secondary:35%, Primary:37%, No education:4% | Mean: 8 years, Range:1-37 years | / |
| Becker HM el ta.  (2015/Canada)  [47] | Mixed-methods (quantitative/qualitative survey) | we aimed to obtain a better understanding of what information patients and their family members would like to have access to in empowering them in disease and life management, and how they perceive the availability and quality of current information, recognizing that the ultimate goal of patient care is to improve QoL. | CD:179, UC:93, IBD unclassified:9, Family members of IBD patients:32 | CD or UC: 65/207 | Range: 0-69 | / | Mean(95% CI): CD:5.9 (4.8–6.9) UC:2.9 (2.0–3.7) | / |
| Pittet V el ta.  (2014/Switzerland)[48] | Cross-sectional (questionnaire) | To examine the association between information seeking activity and treatment compliance among IBD patients. To compare information sources and concerns between compliant and non- compliant patients | CD: 281, UC: 231 | 248/264 | Mean:41  SD: 14 | Primary (mandatory) education level: 11.1%, Secondary education  level: 66.2%, University level:21.9%, Missing value: 0.8% | Median:7 years | / |
| Lesnovska KP  el ta.  (2014/Sweden)  [49] | Qualitative (interview) | To explore the need for knowledge as expressed by patients diagnosed with inﬂammatory bowel disease. | CD:18 UC:12 | 14/16 | Range:29-83 | / | 0–5 years since diagnosis:8, >5–10 years since diagnosis:4, >10–15 years since diagnosis:6, >15 years or more since diagnosis:12 | / |
| Burisch J el ta.  (2014/Denmark)[50] | A multicenter, cross-sectional cohort study (questionnaire) | Our aim was to investigate whether there is a difference between Eastern and Western Europe in health care and education of patients with inflammatory bowel disease (IBD). | CD:363, UC: 485 IBDU: 99 | 519/428 | / | Completed academic education:22.0%, Completed non-academic education: 54.7%, Currently in education: 13.9%, No education: 9.4% | / | / |
| Viazis N el ta.  (2013/Greece)  [51] | Cross-sectional (questionnaire) | The aim of this study was to identify inflammatory bowel disease (IBD) patients’ perspectives regarding everyday life issues. | CD:642, UC:539 | 594/587 | ＜18:42, 18-29:259, 30-39:372, 40-49:270, 50-59:163, ≥60:75 | Basic education: 10%, Secondary education: 42%, University education: 32%, Postgraduate education:16% | ＜1 years: 147, 1-5 years: 425, 6-10 years: 305, 11-15 years: 134, 16-20 years: 82, ＞20 years:88 | Remission:81% |
| Blumenstein I  el ta.  (2013/Germany)[52] | Cross-sectional (questionnaire) | To assess sources of information and patient knowledge in Irish and German inflammatory bowel disease patients. | CD:195, UC:75 | 165/138 | Median:38 | Irish junior certificate/German equivalent: 52%, Irish leaving certificate / German Abitur: 27%, Third level education:21% | Range:5.0-20.5 | / |
| Wong S el ta.  (2012/Canada)  [53] | Cross-sectional (questionnaire) | To survey persons with longstanding IBD as to their information needs and preferred vehicles of information delivery. | CD:132, UC:138, IBD-type unspecified: 1 | 106/165 | Mean:46.5, SD:14.5 | No postsecondary: 35.8%, Trades/non-university certificate or diploma/below bachelor degree: 36.4%, Bachelor degree:13.6%, Above bachelor degree:11.2% | / | Active disease (MIBDI): 52.1% |
| Echarri A el ta.  (2022/Spain)  [54] | a multicenter, observational cross-sectional study (an online questionnaire) | We aim to assess patients’ information habits and patients’ and professionals’ perceptions ofa national website integrated as an educational resource for the IBD unit. | CD:232, UC:166 | 170/229 | Mean: 42.9, SD:11.8 | Elementary: 5.5%, Secondary: 21.1%, Vocational secondary: 42.2%, University:31.2% | <2 years: 84, Between 2 and 8 years: 133, >8 years: 181 | / |
| Cullen G el ta.  (2010/ Ireland)[55] | Cross-sectional (questionnaire) | The aim of this study was to assess attitudes towards and knowledge of medication safety in inflammatory bowel disease (IBD). | CD:56 UC:44 | 51/49 | Median:37, IQR:29-49 | / | Median: 7years [IQR: 3-9] | CD: HBI (mean): 2  UC: Mayo score (mean):1 |
| Pérez-Pérez M el ta.  (2019/Spain)  [56] | Qualitative (analysis of forum posts) | / | / | / | / | / | / | / |

HBI: Harvey–Bradshaw Index, MIBDI: Manitoba IBD Index, SCCAI: simple clinical colitis activity index, CD: Crohn’s disease, UC: ulcerative colitis, IBD: inflammatory bowel disease

/: not reported

**Reference**

1. Pan J, Bian Q, Wang X, Tu Y, Ding W, Xie L. Investigation and analysis of the health education needs in patients with inflammatory bowel disease. Journal of Bengbu Medical College. 2019;44:1687–90.

2. Lin S, Chen Y, Cao Q. Ways of acquiring and needs of requiring disease-related knowledge among patients with inflammatory bowel disease. Chin J Gen Pract. 2018;17(8): 621-625.

3. Gui L, Ye Q, Wang M, Wang M, Zhang Q. A qualitative study on health education needs of patients with inflammatory bowel disease. Practical Clinical Medicine. 2022;23:55–8.

4. Gu J, Xiang Y, Yan S, Zhu L. Analysis on the user behavior of utilizing the information support platform for inflammatory bowel disease. Military Nursing. 2021;38:87–9.

5. Gu J, Lu X, Niu M, Zhu L. Inflammatory bowel disease patient's experience of information seeking: a qualitative research. Chinese Nursing Management. 2016;16:593–6.

6. Bo J, Liu X, Jia J, Wang Y, Chen Y. Self-management needs of patient with inflammatory bowel disease from perspective of informatization：a qualitative research. Military Nursing. 2022;39:21–4.

7. Bernstein KI, Promislow S, Carr R, Rawsthorne P, Walker JR, Bernstein CN. Information needs and preferences of recently diagnosed patients with inflammatory bowel disease. Inflamm Bowel Dis. 2011;17:590–8.

8. Aluzaite K, Greveson K, Ben-Horin S, Leong R, Haj O, Schultz M. Barriers to international travel in inflammatory bowel disease patients. J Travel Med. 2021;28:taaa197.

9. Yu Q, Xu L, Li L, Zhi M, Gu Y, Wang X, et al. Internet and WeChat used by patients with Crohn’s disease in China: a multi-center questionnaire survey. BMC Gastroenterol. 2019;19:97.

10. Sun S, Hu Y, Li H, Chen J, Lou Y, Weng C, et al. Patients’ perspectives on, experience with and concerns about crohn’s disease: insights from Chinese social media. BMC Gastroenterol. 2023;23:105.

11. van Erp LW, Neijenhuis MK, Heida W, Derwig J, Geleijns CE, Groenen MJM, et al. Improving Care for Recently Diagnosed Inflammatory Bowel Disease Patients: Lessons Learned From a Patient-Centred, Mixed-Method Study. J Crohns Colitis. 2022;16:737–45.

12. Karadag P, Morris B, Woolfall K. The information and support needs of patients living with inflammatory bowel disease: A qualitative study. Chronic Illn. 2022;18:356–69.

13. Goren I, Sharvit G, Godny L, Fatal SE, Barkan R, Hag O, et al. Exploring Popular Social Media Networks for Patients With Inflammatory Bowel Diseases: Insights for Better Care. Journal of Clinical Gastroenterology. 2022;56:e203.

14. Dos Santos Marques IC, Herbey II, Theiss LM, Shao CC, Fouad MN, Scarinci IC, et al. Understanding the surgical experience for Black and White patients with inflammatory bowel disease (IBD): The importance of health literacy. The American Journal of Surgery. 2022;223:303–11.

15. Chamorro-de-Vega E, Romero-Jiménez R, Escudero-Vilaplana V, Ais-Larisgoitia A, Lobato Matilla ME, González CM, et al. Information and Communication Technologies in Patients With Immune-Mediated Inflammatory Diseases: Cross-sectional Survey. J Med Internet Res. 2022;24:e37445.

16. Spinelli A, Carvello M, Adamina M, Panis Y, Warusavitarne J, Tulchinsky H, et al. Patients’ perceptions of surgery for inflammatory bowel disease. Colorectal Dis. 2021;23:2690–8.

17. Nachury M, Bouhnik Y, Serrero M, Filippi J, Roblin X, Kirchgesner J, et al. Patients’ real-world experience with inflammatory bowel disease: A cross-sectional survey in tertiary care centres from the GETAID group. Dig Liver Dis. 2021;53:434–41.

18. Long MD, Grewe ME, Cerciello E, Weisbein L, Catabay K, Kappelman MD. A Patient-Prioritized Agenda for Information Needs During the COVID-19 Pandemic: A Qualitative Study of Patients With Inflammatory Bowel Disease. Crohns Colitis 360. 2021;3:otab066.

19. Grunert PC, Reuken PA, Stallhofer J, Teich N, Stallmach A. Inflammatory Bowel Disease in the COVID-19 Pandemic: the Patients’ Perspective. Journal of Crohn’s & colitis. 2020;14.

20. Lee MJ, Jones GL, Lobo AJ, Brown SR, Collaborators the pCD. Survey to define informational needs of patients undergoing surgery for Crohn’s anal fistula. Colorectal Disease. 2021;23:132–44.

21. Cury DB, Paez LEF, Micheletti AC, Reis ST. The Impact of Electronic Media on Patients with Inflammatory Bowel Disease. Risk Manag Healthc Policy. 2021;14:809–13.

22. Cohan JN, Ozanne EM, Hofer RK, Kelly YM, Kata A, Larsen C, et al. Ileostomy or ileal pouch-anal anastomosis for ulcerative colitis: patient participation and decisional needs. BMC Gastroenterol. 2021;21:347.

23. Chowdhary TS, Thompson J, Gayam S. Social Media Use for Inflammatory Bowel Disease in a Rural Appalachian Population. Telemed J E Health. 2021;27:402–8.

24. Aboubakr A, Riggs AR, Jimenez D, Mella MT, Dubinsky MC. Identifying Patient Priorities for Preconception and Pregnancy Counseling in IBD. Dig Dis Sci. 2021;66:1829–35.

25. Yin R, Neyens DM. Online Health Resource Use by Individuals With Inflammatory Bowel Disease: Analysis Using the National Health Interview Survey. J Med Internet Res. 2020;22:e15352.

26. Lee MJ, Marshall JH, Jones GL, Lobo AJ, Brown SR. The informational and decisional preferences of patients undergoing surgery for Crohn’s anal fistula: a qualitative study. Colorectal disease : the official journal of the Association of Coloproctology of Great Britain and Ireland. 2020;22.

27. Khalil C, Van Deen W, Dupuy T, Bonthala N, Almario C, Spiegel B. Developing Patient-Centered Inflammatory Bowel Disease-Related Educational Videos Optimized for Social Media: Qualitative Research Study. JMIR medical education. 2020;6.

28. Keller R, Fusco S, Stange EF, Malek NP, Wehkamp J, Klag T. Infodemiology of Crohn’s disease and Ulcerative colitis using Google Trends - an approach to investigate patient needs. Zeitschrift fur Gastroenterologie. 2020;58.

29. Zigron S, Bronstein J. “Help is where you find it”: The role of weak ties networks as sources of information and support in virtual health communities. Journal of the Association for Information Science and Technology. 2019;70:130–9.

30. Włodarczyk M, Włodarczyk J, Zalewska K, Olczyk M, Maryńczak K, Gajewski P, et al. Preferences of patients with inflammatory bowel disease for receiving specialized health services using technology: the role of Internet and other sources of medical information. Pol Przegl Chir. 2019;91:1–6.

31. Reich J, Guo L, Groshek J, Weinberg J, Chen W, Martin C, et al. Social Media Use and Preferences in Patients With Inflammatory Bowel Disease. Inflammatory bowel diseases. 2019;25.

32. Marrie RA, Walker JR, Graff LA, Patten SB, Bolton JM, Marriott JJ, et al. Gender differences in information needs and preferences regarding depression among individuals with multiple sclerosis, inflammatory bowel disease and rheumatoid arthritis. Patient Educ Couns. 2019;102:1722–9.

33. Daher S, Khoury T, Benson A, Walker JR, Hammerman O, Kedem R, et al. Inflammatory bowel disease patient profiles are related to specific information needs: A nationwide survey. World J Gastroenterol. 2019;25:4246–60.

34. Wu Q, Zhong J. Disease-related information requirements in patients with Crohn’s disease. Patient Prefer Adherence. 2018;12:1579–86.

35. Philip V, Soubieres A, Poullis A. Health concerns associated with travelling with inflammatory bowel disease (IBD): a questionnaire survey. Clin Med (Lond). 2018;18:288–92.

36. McDermott E, Healy G, Mullen G, Keegan D, Byrne K, Guerandel A, et al. Patient Education in Inflammatory Bowel Disease: A Patient-Centred, Mixed Methodology Study. J Crohns Colitis. 2018;12:419–24.

37. Martín Fernández C, Maroto Martín C, Fernández Salazar L. Using the internet to evaluate the opinion of patients with inflammatory bowel disease with regard to the available information. Rev Esp Enferm Dig. 2018;110:274–84.

38. Dibley L, Czuber-Dochan W, Wade T, Duncan J, Burch J, Warusavitarne J, et al. Patient Decision-Making About Emergency and Planned Stoma Surgery for IBD: A Qualitative Exploration of Patient and Clinician Perspectives. Inflamm Bowel Dis. 2018;24:235–46.

39. Selinger CP, Carbery I, Warren V, Rehman AF, Williams CJ, Mumtaz S, et al. The relationship between different information sources and disease-related patient knowledge and anxiety in patients with inflammatory bowel disease. Aliment Pharmacol Ther. 2017;45:63–74.

40. Larsson K, Lööf L, Nordin K. Stress, coping and support needs of patients with ulcerative colitis or Crohn’s disease: a qualitative descriptive study. Journal of Clinical Nursing. 2017;26:648–57.

41. Britt RK. Online Social Support for Participants of Crohn’s and Ulcerative Colitis Groups. Health Commun. 2017;32:1529–38.

42. Baker DM, Lee MJ, Jones GL, Brown SR, Lobo AJ. The Informational Needs and Preferences of Patients Considering Surgery for Ulcerative Colitis: Results of a Qualitative Study. Inflamm Bowel Dis. 2017;24:179–90.

43. Reich J, Guo L, Hall J, Tran A, Weinberg J, Groshek J, et al. A Survey of Social Media Use and Preferences in Patients with Inflammatory Bowel Disease. Inflammatory Bowel Diseases. 2016;22:2678–87.

44. Pittet V, Vaucher C, Maillard MH, Girardin M, de Saussure P, Burnand B, et al. Information Needs and Concerns of Patients with Inflammatory Bowel Disease: What Can We Learn from Participants in a Bilingual Clinical Cohort? PLoS One. 2016;11:e0150620.

45. Yoo Y-S, Cho O-H, Cha K-S. Disease-Related Knowledge and Information Needs Among Inflammatory Bowel Disease Patients in Korea. Gastroenterol Nurs. 2015;38:455–63.

46. Catalán-Serra I, Huguet-Malavés JM, Mínguez M, Torrella E, Paredes JM, Vázquez N, et al. Information resources used by patients with inflammatory bowel disease: Satisfaction, expectations and information gaps. Gastroenterol Hepatol. 2015;38:355–63.

47. Becker HM, Grigat D, Ghosh S, Kaplan GG, Dieleman L, Wine E, et al. Living with inflammatory bowel disease: A Crohn’s and Colitis Canada survey. Can J Gastroenterol Hepatol. 2015;29:77–84.

48. Pittet V, Rogler G, Mottet C, Froehlich F, Michetti P, de Saussure P, et al. Patients’ information-seeking activity is associated with treatment compliance in inflammatory bowel disease patients. Scand J Gastroenterol. 2014;49:662–73.

49. Lesnovska KP, Börjeson S, Hjortswang H, Frisman GH. What do patients need to know? Living with inflammatory bowel disease. J Clin Nurs. 2014;23:1718–25.

50. Burisch J, Vegh Z, Pedersen N, Cuković-Čavka S, Turk N, Kaimakliotis I, et al. Health care and patients’ education in a European inflammatory bowel disease inception cohort: an ECCO-EpiCom study. J Crohns Colitis. 2014;8:811–8.

51. Viazis N, Mantzaris G, Karmiris K, Polymeros D, Kouklakis G, Maris T, et al. Inflammatory bowel disease: Greek patients’ perspective on quality of life, information on the disease, work productivity and family support. Ann Gastroenterol. 2013;26:52–8.

52. Blumenstein I, McDermott E, Keegan D, Byrne K, Ellison M, Doherty G, et al. Sources of information and factual knowledge in Europeans with inflammatory bowel diseases: a cross-cultural comparison between German and Irish patients. J Crohns Colitis. 2013;7:e331-336.

53. Wong S, Walker JR, Carr R, Graff LA, Clara I, Promislow S, et al. The information needs and preferences of persons with longstanding inflammatory bowel disease. Can J Gastroenterol. 2012;26:525–31.

54. Echarri A, Pérez-Calle JL, Calvo M, Molina G, Sierra-Ausín M, Morete-Pérez MC, et al. Should Inflammatory Bowel Disease Clinicians Provide Their Patients with e-Health Resources? Patients’ and Professionals’ Perspectives. Telemedicine and e-Health. 2022. https://doi.org/10.1089/tmj.2022.0425.

55. Cullen G, Donnellan F, Long S, Forry M, Murray FE. Perceptions of medication safety among patients with inflammatory bowel disease. Scand J Gastroenterol. 2010;45:1076–83.

56. Pérez-Pérez M, Pérez-Rodríguez G, Fdez-Riverola F, Lourenço A. Using Twitter to Understand the Human Bowel Disease Community: Exploratory Analysis of Key Topics. J Med Internet Res. 2019;21:e12610.
